# The impact of mindfulness intervention on negative emotions and quality of life in malignant tumor patients: a systematic review and meta-analysis

**DOI:** 10.3389/fpsyg.2024.1443516

**Published:** 2024-09-18

**Authors:** Zhang Li, Dong Lei, Li Ting, Ran Yao, Wu Jing, Mi Na

**Affiliations:** The Second Affiliated Hospital of Army Medical University, Xinqiao Hospital, Shapingba, China

**Keywords:** mindfulness intervention, malignant tumor, anxiety, depression, quality of life

## Abstract

**Objective:**

This study aims to assess the effect of mindfulness intervention on negative emotions (anxiety and depression) and quality of life in malignant tumor patients.

**Methods:**

The databases, including CNKI, VIP, Wanfang, Chinese Biomedical Literature Database disc (CBMdisc), PubMed, Embase, Cochrane Library, and Web of Science (WoS), were searched from inception to January 2024. Randomized controlled trials examining the effects of mindfulness intervention on negative emotions and quality of life in malignant tumor patients were selected. Meta-analysis was conducted using RevMan 5.1.

**Results:**

A total of 11 studies involving 993 patients were included. Compared with usual care, mindfulness intervention effectively reduced anxiety [*SMD* = −0.81, 95% CI (−1.01, −0.60), *p* < 0.00001], depression [*SMD* = −0.86, 95% CI (−1.01, −0.70), *p* < 0.00001], and improved patients’ quality of life [*SMD* = 0.64, 95% CI (0.50, 0.78), *p* < 0.00001].

**Conclusion:**

Mindfulness intervention can effectively alleviate negative emotions such as anxiety and depression in malignant tumor patients and positively impact their quality of life.

## 1 Introduction

In recent years, the global incidence and mortality rates of malignant tumors have been on the rise, with rates in China surpassing the global average (Cao and Chen, 2019; Ferlay et al., 2020). Malignant tumors not only pose a severe threat to human health but also present significant physical and psychological challenges to patients. Malignant tumor patients often endure pain, weight loss, and a reduced lifespan. Combined with the financial strain of treatment, these symptoms frequently lead to negative emotions such as anxiety and depression, profoundly impacting their quality of life (Lv, 2016). With the deepening of psychological and medical research, the role of positive thinking intervention in chronic disease management has been widely recognized (Oberoi et al., 2020; Bower and Irwin, 2016). For patients with malignant tumors, in addition to physical suffering, psychological stress and negative emotions are common problems. Anxiety, depression, feelings of helplessness, and fear of the future often cause patients’ quality of life to be seriously impaired. And these negative emotions not only weaken patients’ psychological resilience, but may also adversely affect the immune system, thus further affecting the progress of the disease and treatment outcome (Haller et al., 2017). Positive thinking intervention, as a kind of experience-centered psychotherapy, aims to alleviate psychological stress by guiding patients to focus on the present moment and reduce excessive thoughts about the past or future (Johns et al., 2015). Mindfulness interventions include Mindfulness-Based Stress Reduction (MBSR), Mindfulness-Based Cognitive Therapy (MBCT), and Mindfulness Meditation (MBSR), Mindfulness-Based Cognitive Therapy (MBCT), and mindfulness meditation. These interventions help patients learn to live with their pain and reduce overreaction to pain and other uncomfortable symptoms by non-judgmentally observing and accepting inner emotions and feelings (Garland et al., 2019). Studies have shown that positive thinking interventions can significantly reduce anxiety and depressive symptoms in oncology patients, as well as improve their psychological adjustment and life satisfaction (Oberoi et al., 2020; Haller et al., 2017; Johns et al., 2015).

In addition, positive thinking intervention can enhance patients’ physical health by regulating the stress response. It has been found that positive thinking exercises can reduce inflammatory markers and improve immune function in patients, which may have a role in delaying tumor progression (Oberoi et al., 2020; Bower and Irwin, 2016). It is worth noting that positive thinking intervention not only has a positive impact on mental health, but also improves patients’ self-management ability and helps them to better cope with the challenges of daily life, thus improving their quality of life overall (Garland et al., 2019). Based on these findings, more and more clinical trials have included positive thinking intervention as an adjuvant treatment for patients with malignant tumors. In this paper, Meta-analysis was used to further clarify the effectiveness of positive thinking intervention in reducing negative emotions and improving the quality of life of patients with malignant tumors, providing a more solid evidence-based medical basis for clinical practice.

## 2 Materials and methods

The study strictly followed the guidelines of the Cochrane Handbook for Systematic Evaluation of Interventions for systematic evaluation and the article was written in accordance with the PRISMA guidelines (see Supplementary File 1). The review has been registered on the PROSPERO platform under the registration number CRD42024578673.

### 2.1 Literature retrieval

Computer searches were conducted in databases including CNKI, VIP, Wanfang, Chinese Biomedical Literature Database disc (CBMdisc), PubMed, Embase, Cochrane Library, Web of Science (WoS), and others. The search period ranged from the establishment of the databases to January 2024. The search strategy combined subject headings and free terms. For Chinese databases such as CNKI, the search string was SU% = (‘mindfulness’ + ‘mindfulness meditation’ + ‘mindfulness therapy’ + ‘mindfulness stress reduction’ + ‘mindfulness training’ + ‘mindfulness intervention’ + ‘mindfulness cognitive therapy’ + ‘mindfulness practice’) and (‘malignant tumor’ + ‘cancer’ + ‘tumor’) and (‘quality of life’ + ‘life quality’ + ‘quality of survival’ + ‘life quality’) and (‘anxiety’ + ‘depression’ + ‘negative emotions’ + ‘negative mood’). For English databases like PubMed, a combination of subject headings and free terms was used for the search: (Mindfulness or mindfulness meditation or insight meditation or mindfulness based stress reduction or mindfulness based cognitive therapy or MBSR) and (Malignant tumor or malignancies or cancer) and (quality of life) and (anxiety or depression). The complete search strategies for each database are included in Supplementary File 2.

### 2.2 Inclusion and exclusion criteria

#### 2.2.1 Literature inclusion criteria: inclusion was eligible if the following criteria were met

P (Population): Patients with malignant tumors aged ≥18 years, regardless of tumor type and stage.

I (Intervention): Positive thinking intervention (including breathing training, positive thinking meditation, body scanning, positive thinking yoga, etc.).

C (Comparison): Patients in the control group received conventional interventions (such as health education, psychological support, social and family support, etc.).

O (Outcome): Anxiety, depression, quality of life.

S (Study Design): Randomized controlled trial.

#### 2.2.2 Exclusion criteria

In this study, patients who are receiving, or plan to receive during the study period, anxiolytic medication, antidepressant medication or other psychotropic medication will be excluded. This was to ensure that the effects of the study intervention were not confounded by medication, and thus to more accurately assess the independent effects of the orthostatic intervention on anxiety, depression and quality of life. Duplicate publications; publications with inconsistent outcome metrics; publications in the form of abstracts, reviews, case studies, etc.; non-Chinese and English language publications; and publications for which full text or complete data were not available.

### 2.3 Indicators for observation

(1) Primary indicators: Quality of Life Questionnaire-Core 36 (QLQ-C30), the 36-Item Short Form Health Survey (SF-36), the Functional Assessment of Cancer Therapy (FACT), the Self-Rating Anxiety Scale (SAS), the Self-Rating Depression Scale (SDS), the Hospital Anxiety and Depression Scale (HADS). (2) Secondary Indicators: Beck Anxiety Inventory (BAI), Beck’s Depression Inventory (BDI), Pittsburgh Sleep Quality Index (PSQI), Visual Analog Scale (VAS).

### 2.4 Literature selection and quality assessment

#### 2.4.1 Literature screening

Two researchers trained in the field independently conducted literature searches and screenings in designated databases, following predefined search strategies and criteria. Search results were compiled in an EndNote library, and duplicates were removed. In cases of discrepancies during literature screening, a third-party arbitration team was consulted to reach a consensus. Initial screening was based on titles and abstracts; subsequent full-text reading enabled final literature selection adhering to inclusion and exclusion criteria. Information from selected literature, including author(s), publication year, sample size, intervention methods, assessment tools, outcome measures, and conclusions, was extracted using a custom form. Continuous variable data underwent standard deviation conversion and calculation.

#### 2.4.2 Literature quality assessment

Two trained researchers independently evaluated the quality of the included literature across seven dimensions, following the randomized controlled trials (RCTs) quality assessment standards of Cochrane Handbook Assessment criteria included random sequence generation, allocation concealment, blinding of participants and researchers, blinding of outcome assessment, completeness of outcome data, selective outcome reporting, and other potential biases. After an independent quality assessment, results from the two researchers were compared, and discrepancies were resolved through discussion or by seeking arbitration from a third party.

#### 2.4.3 Statistical analysis

For eligible RCT literature, statistical analysis was conducted using the Meta-analysis module in RevMan 5.1. Risk ratios (RR) and 95% confidence intervals (CI) were used for count data. The *I*^2^ statistic and *p* value were employed to assess heterogeneity among results. If *p* ≥ 0.1 and *I^2^* < 50%, indicative of no significant heterogeneity, a fixed-effects model was applied for meta-analysis. Conversely, if *p* < 0.1 and *I^2^* ≥ 50%, indicative of significant heterogeneity, a random-effects model was used. Subgroup analysis was performed to explore sources of heterogeneity. For continuous variables, weighted mean differences (WMD) and 95% CI were employed if the same measurement tool was used; otherwise, standardized mean differences (SMD) and 95% CI were employed.

## 3 Results

### 3.1 Search results

**Figure 1:**
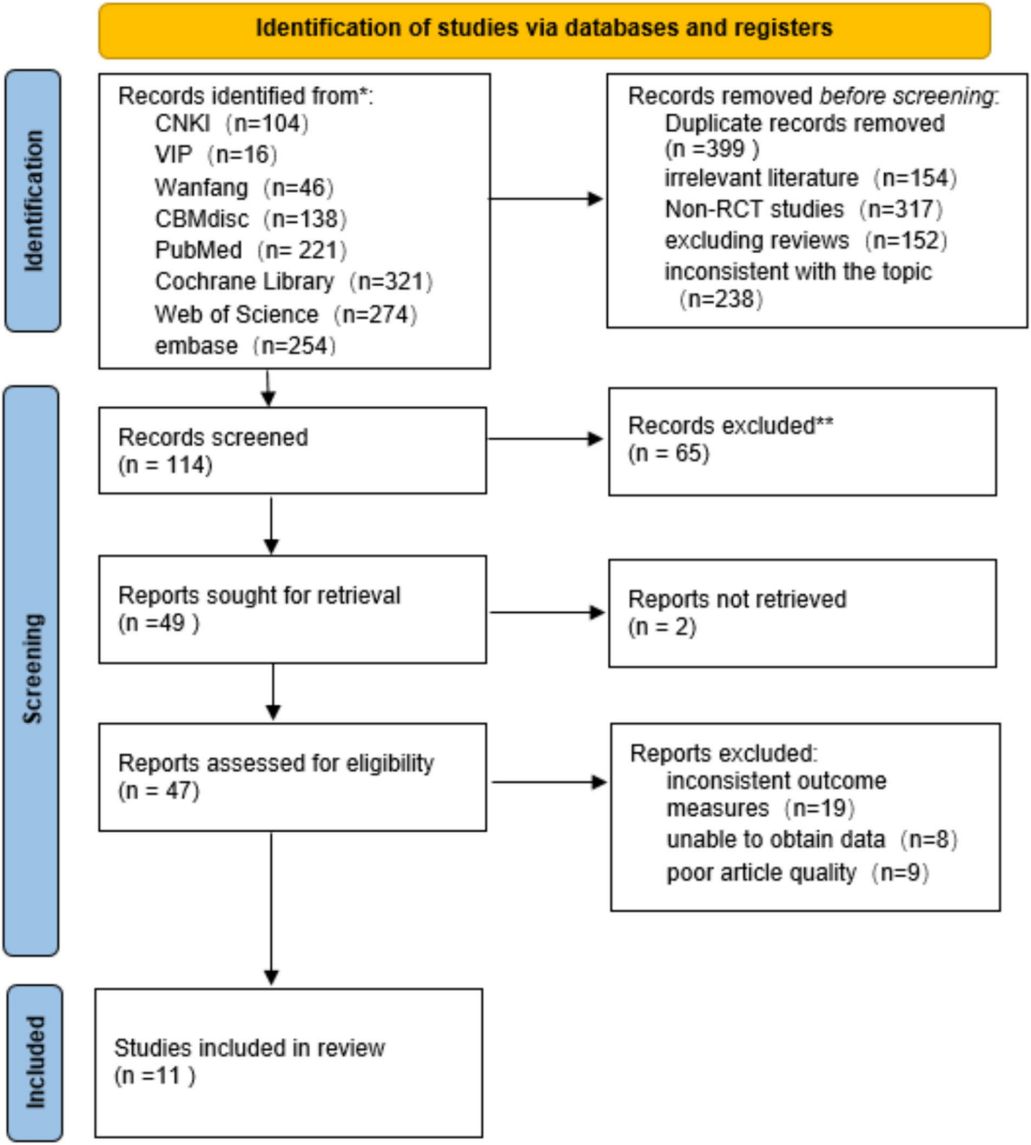
PRISMA flow diagram.

The systematic search initially retrieved a total of 1,301 articles, including 304 Chinese articles and 997 English articles. After thorough reading and screening of the full texts, a total of 11 articles were included (Zhang et al., 2018; Chen, 2021; Hao et al., 2019; Pan et al., 2022; Li et al., 2019; Qing and Gui, 2018; Li et al., 2021; Park et al., 2020; Liu et al., 2019; Zhu et al., 2023; McCombie et al., 2023) (4 in English and 7 in Chinese). The literature screening process is illustrated in Figure 1 (Supplementary File 3).

### 3.2 Basic information and quality assessment of included literature

The 11 included articles (Zhang et al., 2018; Chen, 2021; Hao et al., 2019; Pan et al., 2022; Li et al., 2019; Qing and Gui, 2018; Li et al., 2021; Park et al., 2020; Liu et al., 2019; Zhu et al., 2023; McCombie et al., 2023), encompassing both Chinese and English literature, involved a total sample size of 993 cases, with 496 patients in the intervention group and 497 patients in the control group. Published between 2018 and 2023, these studies had intervention durations ranging from 6 to 12 weeks. For detailed basic information on the included studies, please refer to Table 1 (Supplementary File 4).

**Table 1:** Overview of included literature.

Included studies	Publication date	Type of tumor	Sample size	Intervention measures		Implementation personnel	Duration of intervention	Evaluation time	Outcome measures	Assessment tools
			Control group/observation group	Control group	Observation group					
Zhang et al. (2018)	2018	Lymphoma	45/45	Usual care	Usual Care+Mindfulness-Based Stress Reduction Combined with Aerobic Exercise Training	Psychologists	6 weeks, 6 times/week, 1 time/60 min	Baseline, 6 weekends after intervention	Anxiety, Depression, Quality of Life, Pressure, Subjective Well-being, Expectation Level	SAS, SDS, SF-36, CPSS, MUNSH
Chen (2021)	2021	Lymphoma	38/38	Usual care	Usual Care+Rational Emotive Behavior Therapy Combined with Mindfulness Meditation	Doctors, nurses, and psychological counselors	8 weeks, 2 times/week, 1 time/40 min	Baseline, 8 weekends after intervention	Anxiety, Depression, Quality of Life, Self-Acceptance	SAS, SDS, FACT-B, SAQ
Hao et al. (2019)	2019	Lymphoma	65/65	Usual care	Usual Care+Group Mindfulness Cognitive Therapy	Nurse	8 weeks, 2 times/week, 1 time/1 h	Baseline, 8 weekends after intervention	Anxiety, Depression, Quality of Life	SAS, SDS, QLQ-C30
Pan et al. (2022)	2022	Nasopharyngeal carcinoma NPC	25/25	Usual Care+psychological nursing	Usual Care+psychological nursing+Mindfulness-Based Stress Reduction	Professional trained nurses	8 weeks, 4–5 times/week, 1 time/45 min	Baseline, 8 weekends after intervention	Anxiety, Depression, Quality of Life, pain	HADS, VAS, FACT-H&N
Li et al. (2019)	2019	Gynecologic malignancy	40/40	Usual care	Usual Care+Mindfulness-Based Stress Reduction	Professional trained nurses	8 weeks, once a week, 1 time/2 ~ 3 h	Baseline, 8 weekends after intervention	Anxiety, Depression, Quality of Life, Pressure, Self-assessment of symptoms	SAS, SDS, SF-36, CPSS
Qing and Gui (2018)	2018	Cervix	48/48	Usual care	Usual Care+Mindfulness-Based Stress Reduction	Professional trained nurses	8 weeks, once a week, 1 time/3 h	Baseline, 8 weekends after intervention	Anxiety, Depression, Quality of Life, Fatigue, sleep quality,	SAS, SDS, FACT-CX, PSQI
Li et al. (2021)	2021	Nasopharyngeal carcinoma NPC	63/63	Usual care	Usual Care+Mindfulness-Based Stress Reduction	Psychologists	8 weeks, once a week, 1 time/30 min	Baseline, 8 weekends after intervention	Anxiety, Depression, Quality of Life, Pressure, Self-assessment of symptoms	BAI, BDI, QLQ-C30, CPSS
Park et al. (2020)	2020	Lymphoma	36/38	Usual care	Usual Care+Mindfulness Cognitive Therapy	Psychologists, psychiatrists, nurses	8 weeks, once a week, 1 time/2 h	Baseline, 8 weeks after intervention, and 12 weeks after intervention	Anxiety, Depression, Quality of Life, wearily, mental health, Fear of Cancer Recurrence	HADS, FACT-G, CARS, BFI, FACIT-Sp
Liu et al. (2019)	2019	Thyroid cancer	53/49	Usual care	Usual Care+Mindfulness-Based Stress Reduction	Psychologists	8 weeks, once a week	Baseline, 8 weekends after intervention, and at the end of 3 months after intervention	Anxiety, Depression, Quality of Life, wearily	SAS, SDS, QLQ-C30
Zhu et al. (2023)	2023	Lymphoma	51/50	Usual care	Mindfulness-Based Stress Reduction	Professional trainers	8 weeks, once a week, 1 time/2 h	Baseline, 8 weekends after intervention	Anxiety, Depression, Quality of Life, Cognitive Emotion	SAS, SDS, FACT-B
McCombie et al. (2023)	2023	Rectum	33/35	Psychological Education+Cognitive Behavioral Skills and Support	Mindfulness intervention	Psychologist	8 weeks, once a week, 1 time/2 h	Baseline, 8 weekends after intervention, and at the end of 6 months after intervention	Anxiety, Depression, Quality of Life	HADS, QOL

QLQ-C30, quality of life questionnaire-core 30; SF-36, the 36- item Short From Health Survey; FACT, Functional Assessment of Cancer Therapy; SAS, self-rating anxiety scale; SDS, self-rating depression scale; HADS, hospital anxiety and depression scale; BAI, Beck Anxiety Inventory; BDI, Beck’s Depression Inventory; PSQI, Pittsburgh sleep quality index; VAS, visual analog scale; CPSS, Chinese Version Perceived Stress Scale.

### 3.3 Risk of bias

**Figure 2:**
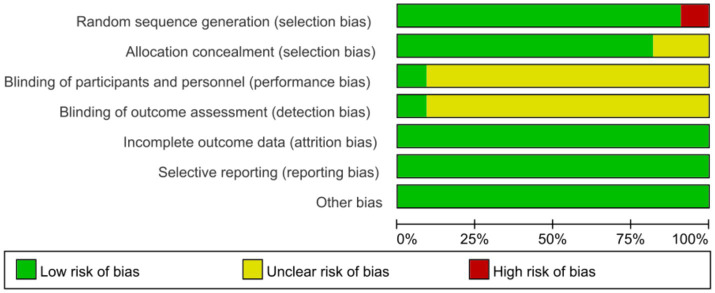
Risk preference diagram.

**Figure 3:**
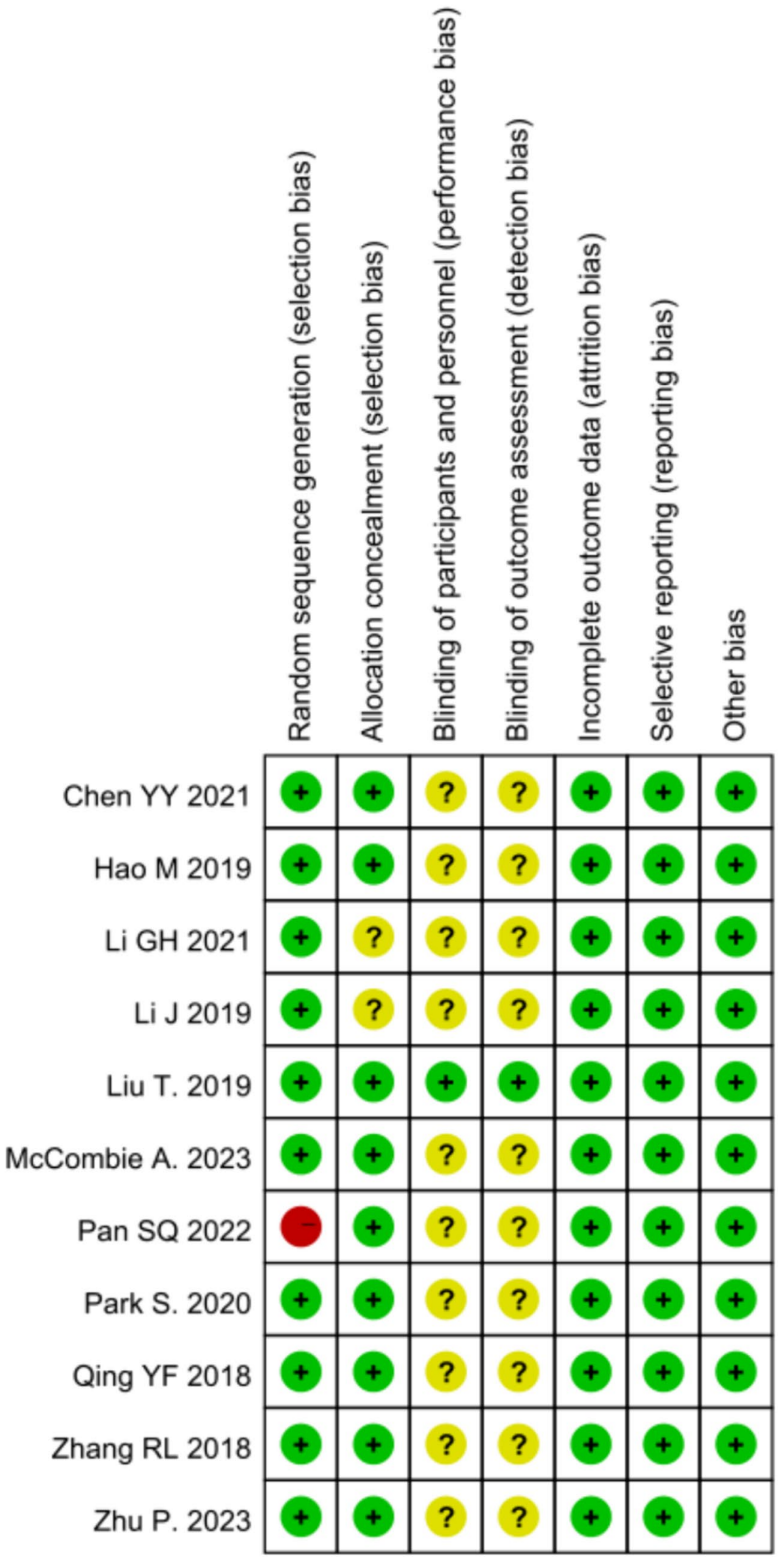
A summary of risk preference.

All 11 studies (Zhang et al., 2018; Chen, 2021; Hao et al., 2019; Pan et al., 2022; Li et al., 2019; Qing and Gui, 2018; Li et al., 2021; Park et al., 2020; Liu et al., 2019; Zhu et al., 2023; McCombie et al., 2023) used random number method to divide subjects into experimental and control groups, which is low risk of bias; 2 studies (Chen, 2021; Hao et al., 2019; Pan et al., 2022) did not describe allocation concealment, which is potentially at risk of bias; due to the nature of psychological interventions, they usually cannot be fully blinded, and 10 studies (Zhang et al., 2018; Chen, 2021; Hao et al., 2019; Pan et al., 2022; Li et al., 2019; Li et al., 2021; Park et al., 2020; Liu et al., 2019; Zhu et al., 2023; McCombie et al., 2023) did not describe blinding; 11 studies (Zhang et al., 2018; Chen, 2021; Hao et al., 2019; Pan et al., 2022; Li et al., 2019; Qing and Gui, 2018; Li et al., 2021; Park et al., 2020; Liu et al., 2019; Zhu et al., 2023; McCombie et al., 2023) described lost to visit numbers; all 11 studies (Zhang et al., 2018; Chen, 2021; Hao et al., 2019; Pan et al., 2022; Li et al., 2019; Qing and Gui, 2018; Li et al., 2021; Park et al., 2020; Liu et al., 2019; Zhu et al., 2023; McCombie et al., 2023) used intention-to-treat analyses; see Figures 2, 3 for the quality of evidence, among others, according to the grading of assessments and evaluations.

### 3.4 Meta-analysis results

#### 3.4.1 Impact of mindfulness intervention on anxiety in malignant tumor patients

**Figure 4:**
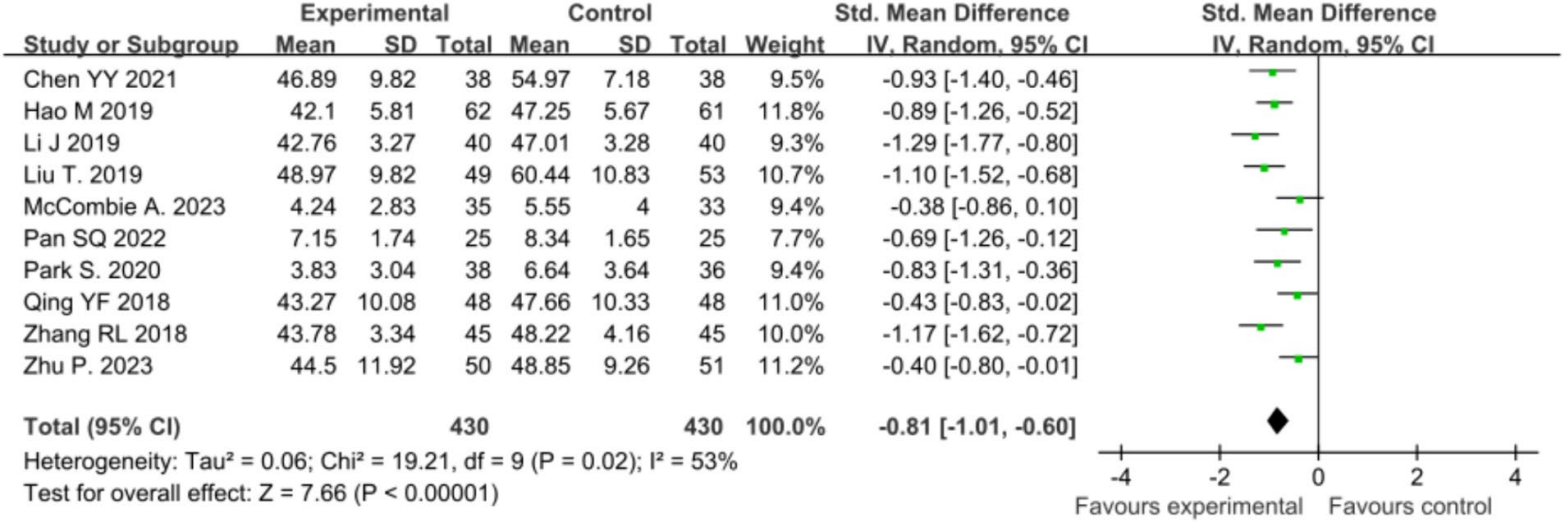
Sensitivity analysis comparing post-intervention anxiety between the two patient groups.

Eleven studies (Zhang et al., 2018; Chen, 2021; Hao et al., 2019; Pan et al., 2022; Li et al., 2019; Qing and Gui, 2018; Li et al., 2021; Park et al., 2020; Liu et al., 2019; Zhu et al., 2023; McCombie et al., 2023) investigated the effect of mindfulness intervention on anxiety. Meta-analysis revealed significant heterogeneity among studies (*p* < 0.0001, *I^2^* = 92%), requiring the use of a random-effects model. The analysis indicated a significant difference in anxiety scores between the intervention and control groups post-intervention [*SMD* = −0.60, 95% CI (−1.08, −0.11), *p* = 0.02]. Sensitivity analysis revealed a decrease in heterogeneity (*p* = 0.02, *I^2^* = 53%) upon excluding the study by Li et al. (2021). This may be related to the frequency and short duration of interventions. Further analysis excluding this study along with the remaining 10 studies (Zhang et al., 2018; Chen, 2021; Hao et al., 2019; Pan et al., 2022; Li et al., 2019; Qing and Gui, 2018; Park et al., 2020; Liu et al., 2019; Zhu et al., 2023; McCombie et al., 2023) showed a persistent, significant difference in anxiety scores between the two groups post-intervention [*SMD* = −0.81, 95% CI (−1.01, −0.60), *p* < 0.00001], as illustrated in Figure 4.

#### 3.4.2 Influence of mindfulness intervention on depression in malignant tumor patients

**Figure 5:**
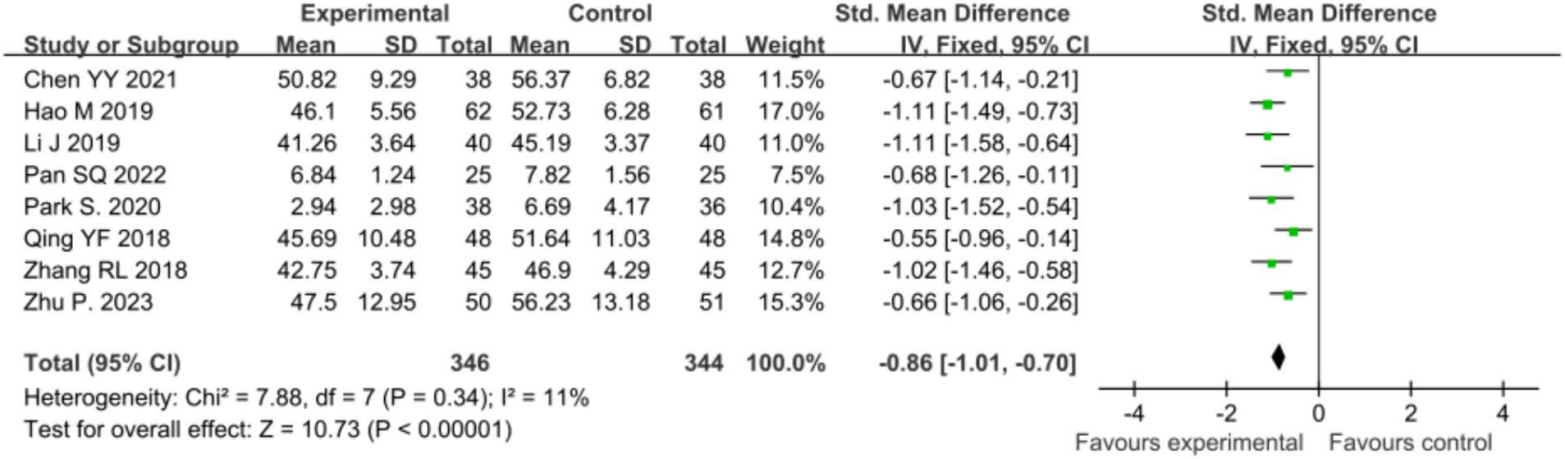
Sensitivity analysis comparing post-intervention anxiety between the two patient groups.

Eleven studies (Zhang et al., 2018; Chen, 2021; Hao et al., 2019; Pan et al., 2022; Li et al., 2019; Qing and Gui, 2018; Li et al., 2021; Park et al., 2020; Liu et al., 2019; Zhu et al., 2023; McCombie et al., 2023) examined the effects of mindfulness intervention on depression in malignant tumor patients. Meta-analysis revealed high heterogeneity (*p* < 0.00001, *I^2^* = 94%), necessitating analysis using a random-effects model, which demonstrated significantly lower depression scores in the intervention group compared to the control group [*SMD* = −0.59, 95% CI (−1.16, −0.02), *p* = 0.004]. Sensitivity analysis was conducted to explore sources of high heterogeneity by sequentially excluding studies and reanalyzing the remaining ones. Upon exclusion of the study by Li et al. (2021), heterogeneity decreased (*p* = 0.002, *I^2^* = 65%); exclusion of the study by McCombie et al. (2023) resulted in *p* = 0.04, *I^2^* = 50%; exclusion of the study by Liu et al. (2019) led to *p* = 0.05, *I^2^* = 49%. After excluding these three studies, heterogeneity significantly decreased to *I^2^* = 11%, suggesting that these studies might be the main source of heterogeneity, possibly due to differences in assessment tools or types of diseases. Following the exclusion of the studies by Li et al. (2021), McCombie et al. (2023), and Liu et al. (2019), a fixed-effects model was used to meta-analyze the remaining eight studies (Zhang et al., 2018; Chen, 2021; Hao et al., 2019; Pan et al., 2022; Li et al., 2019; Qing and Gui, 2018; Park et al., 2020; Zhu et al., 2023), revealing a statistically significant difference in depression scores [*SMD* = −0.86, 95% CI (−1.01, −0.70), *p* < 0.00001], indicating a notable improvement in depression after mindfulness intervention. This result is more robust as heterogeneity significantly decreased, enhancing the credibility of the findings, as illustrated in Figure 5.

#### 3.4.3 Impact of mindfulness intervention on quality of life in malignant tumor patients

**Figure 6:**
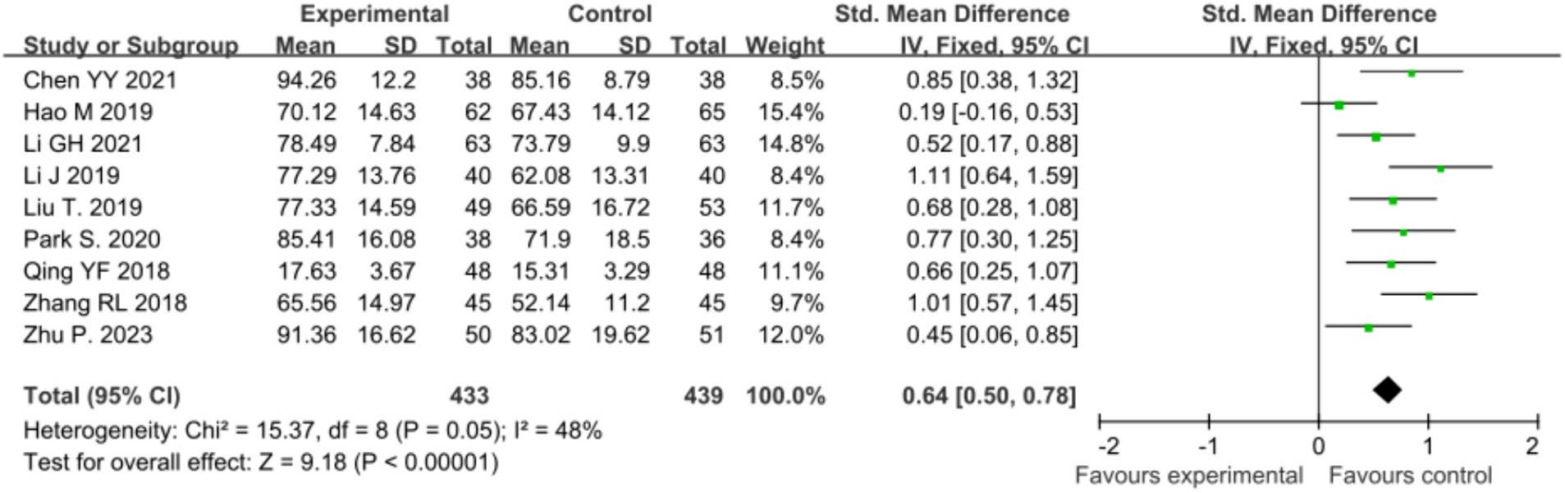
Sensitivity analysis comparing post-intervention quality of life between the two patient groups.

Ten studies (Zhang et al., 2018; Chen, 2021; Hao et al., 2019; Pan et al., 2022; Li et al., 2019; Qing and Gui, 2018; Li et al., 2021; Park et al., 2020; Liu et al., 2019; Zhu et al., 2023; McCombie et al., 2023) reported a positive impact of mindfulness intervention on the quality of life in malignant tumor patients. The results showed high heterogeneity (*p* < 0.00001, *I^2^* = 89%), requiring meta-analysis using a random-effects model. The analysis revealed a statistically significant difference in quality of life scores between the two groups post-mindfulness intervention [*SMD* = 0.98, 95% CI (0.56, 1.40), *p* < 0.00001], indicating a significantly higher quality of life in the mindfulness intervention group compared to the control group. Sensitivity analysis was conducted to explore and mitigate heterogeneity by sequentially excluding studies. After excluding the study by Pan et al. (2022) heterogeneity testing resulted in *p* = 0.005, *I^2^* = 48%, suggesting that this study may have a significant impact on overall heterogeneity. Analysis suggested that the source of heterogeneity may be related to differences in specific modules within the assessment tools. Following exclusion of the study by Pan et al. (2022), a fixed-effects model was used to meta-analyze the remaining nine studies (Zhang et al., 2018; Chen, 2021; Hao et al., 2019; Li et al., 2019; Qing and Gui, 2018; Li et al., 2021; Park et al., 2020; Liu et al., 2019; Zhu et al., 2023), revealing a significant effect of mindfulness intervention on improving the quality of life in malignant tumor patients [*SMD* = 0.64, 95% CI (0.50, 0.78), *p* < 0.00001], as illustrated in Figure 6.

### 3.5 Subgroup analysis

Due to the heterogeneity of the included studies, this study conducted subgroup analyses by exploring different assessment tools, different types of positive thinking interventions, and the effects of positive thinking interventions on different tumor types, the results of which are summarized below (Supplementary File 5).

#### 3.5.1 Impact on anxiety

Seven studies (Zhang et al., 2018; Chen, 2021; Hao et al., 2019; Li et al., 2019; Qing and Gui, 2018; Liu et al., 2019; Zhu et al., 2023) used the SAS scale and the results showed that the positive thinking intervention group had significantly lower anxiety scores than the control group with moderate heterogeneity (*I*^2^ = 58%, *p* < 0.00001). In addition, 3 studies (Pan et al., 2022; Park et al., 2020; McCombie et al., 2023) used the HADS scale and the results similarly showed that the positive thinking intervention group had lower anxiety scores than the control group with low heterogeneity (*I*^2^ = 38%, *p* = 0.0009). In addition, 1 study (Li et al., 2021) used the BAI scale to assess anxiety and the results showed that the positive thinking intervention group also had lower scores than the control group (*p* < 0.00001).

According to the different types of positive thinking interventions, 2 studies (Hao et al., 2019; Park et al., 2020) used positive thinking cognitive therapy to alleviate patients’ anxiety, and the results showed that the anxiety scores of the positive thinking intervention group were significantly lower than those of the control group with no heterogeneity (*I*^2^ = 0%, *p* < 0.00001). While 8 studies (Zhang et al., 2018; Pan et al., 2022; Li et al., 2019; Qing and Gui, 2018; Li et al., 2021; Liu et al., 2019; Zhu et al., 2023; McCombie et al., 2023) used Positive Mindfulness Stress Reduction Therapy and the results showed that there was no statistically significant difference in anxiety scores between Positive Mindfulness Intervention Group and Control Group (*I*^2^ = 94%, *p* = 0.14) 0.1 study (Chen, 2021) used Positive Mindfulness Meditation to alleviate patients’ anxiety and the results showed that the Positive Mindfulness Intervention Group had significantly lower scores than the Control Group (*p* = 0.0001).

There were also significant differences in the effect of positive thinking intervention on anxiety in patients with different tumor types. Five studies (Zhang et al., 2018; Chen, 2021; Hao et al., 2019; Park et al., 2020; Zhu et al., 2023) reported that anxiety in breast cancer patients was effectively alleviated with positive thinking intervention (*I*^2^ = 42%, *p* < 0.00001). However, two studies (Pan et al., 2022; Li et al., 2021) showed no significant effect of positive thinking intervention on anxiety scores in nasopharyngeal cancer patients (*I*^2^ = 97%, *p* = 0.71). Similarly, two studies (Li et al., 2019; Qing and Gui, 2018) reported no statistically significant effect of positive thinking intervention on anxiety scores in gynecological oncology patients (*I*^2^ = 86%, *p* = 0.05). In contrast, 1 study (Liu et al., 2019) showed that positive thinking intervention had a significant mitigating effect on anxiety in thyroid cancer patients (*p* < 0.00001). Finally, one study (McCombie et al., 2023) reported no significant effect of positive thinking intervention on anxiety scores of rectal cancer patients (*p* = 0.13).

#### 3.5.2 Impact on depression

Seven studies (Zhang et al., 2018; Chen, 2021; Hao et al., 2019; Li et al., 2019; Qing and Gui, 2018; Liu et al., 2019; Zhu et al., 2023) used the SDS scale to assess depressed mood, and the results showed that the positive thinking intervention group had significantly lower depression scores than the control group, but with higher heterogeneity (*I*^2^ = 84%, *p* < 0.00001). In addition, three studies (Pan et al., 2022; Park et al., 2020; McCombie et al., 2023) assessed using the HADS scale and showed no statistically significant difference in depression scores between the positive thinking intervention group and the control group (*I*^2^ = 81%, *p* = 0.05). In addition, 1 study (Qing and Gui, 2018) assessed depression using the BDI scale and showed that the positive thinking intervention group had significantly lower scores than the control group (*p* < 0.00001).

According to the different types of positive thinking interventions, two studies (Hao et al., 2019; Park et al., 2020) used positive thinking cognitive therapy to alleviate patients’ depression, and the results showed that the depression scores of the positive thinking intervention group were significantly lower than those of the control group with no heterogeneity (*I*^2^ = 0%, *p* < 0.00001). However, eight studies (Zhang et al., 2018; Pan et al., 2022; Li et al., 2019; Qing and Gui, 2018; Li et al., 2021; Liu et al., 2019; Zhu et al., 2023; McCombie et al., 2023) used Positive Mindfulness Stress Reduction Therapy and showed no statistically significant difference in depression scores between Positive Mindfulness Intervention Group and Control Group (*I*^2^ = 94%, *p* = 0.14). One study (Chen, 2021) used Positive Mindfulness Meditation to alleviate patients’ depressive mood and showed that Positive Mindfulness Intervention Group scored significantly lower than Control Group (*p* = 0.004).

Positive thinking intervention also produced significant differences in depression in patients with different tumor types. Five studies (Zhang et al., 2018; Chen, 2021; Hao et al., 2019; Park et al., 2020; Zhu et al., 2023) reported significant relief of depression in breast cancer patients with positive thinking intervention without heterogeneity (*I*^2^ = 0%, *p* < 0.00001). However, two studies (Pan et al., 2022; Li et al., 2021) showed no significant effect of positive thinking intervention on depression scores in nasopharyngeal cancer patients (*I*^2^ = 98%, *p* = 0.62). In contrast, two studies (Li et al., 2019; Qing and Gui, 2018) reported a significant mitigating effect of positive thinking intervention on depression in gynecological oncology patients (*I*^2^ = 68%, *p* = 0.004). In contrast, one study (Liu et al., 2019) showed that positive thinking intervention also had a significant alleviating effect on depression in thyroid cancer patients (*p* < 0.00001). Finally, one study (McCombie et al., 2023) reported no significant effect of positive thinking intervention on depression scores in rectal cancer patients (*p* = 0.54).

#### 3.5.3 Impact on quality of life

Five studies (Chen, 2021; Pan et al., 2022; Qing and Gui, 2018; Park et al., 2020; Zhu et al., 2023) used the FACT scale and the results showed that the quality of life scores of the positive thinking intervention group were better than those of the control group, but with a high degree of heterogeneity (*I*^2^ = 94%, *p* = 0.0009). In addition, three studies (Hao et al., 2019; Li et al., 2021; Park et al., 2020) assessed quality of life using the QLQ-C30 scale, and the results similarly showed that the positive thinking intervention group had better scores than the control group with moderate heterogeneity (*I*^2^ = 53%, *p* = 0.005). In addition, two studies (Zhang et al., 2018; Hao et al., 2019) assessed quality of life using the SF-36 scale and showed that the positive thinking intervention group scored significantly better than the control group with no heterogeneity (*I*^2^ = 0%, *p* < 0.00001).

According to the different types of positive thinking interventions, two studies (Hao et al., 2019; Park et al., 2020) used positive thinking cognitive therapy to improve patients’ quality of life, and the results showed that there was no statistically significant difference between the positive thinking intervention group and the control group (*I*^2^ = 74%, *p* = 0.12). However, seven studies (Zhang et al., 2018; Pan et al., 2022; Li et al., 2019; Qing and Gui, 2018; Li et al., 2021; Liu et al., 2019; Zhu et al., 2023) used positive thinking stress reduction therapy, and the results showed that the positive thinking intervention group was effective in improving patients’ quality of life, but with high heterogeneity (I^2^ = 92%, *p* = 0.14). In addition, one study (Chen, 2021) used positive thinking meditation to improve patients’ quality of life, and the results showed that the scores of the positive thinking intervention group were significantly better than those of the control group (*p* = 0.004).

There were significant differences in the impact of positive thinking intervention on quality of life of patients with different tumor types. Five studies (Zhang et al., 2018; Chen, 2021; Hao et al., 2019; Park et al., 2020; Zhu et al., 2023) reported significant improvement in quality of life of breast cancer patients with positive thinking intervention (*I*^2^ = 63%, *p* < 0.0001). On the contrary, two studies (Pan et al., 2022; Li et al., 2021) showed no significant effect of positive thinking intervention on the quality of life of nasopharyngeal cancer patients (*I*^2^ = 98%, *p* = 0.25). In addition, two studies (Li et al., 2019; Qing and Gui, 2018) reported a significant improvement effect of positive thinking intervention on the quality of life of gynecological oncology patients (*I*^2^ = 50%, *p* = 0.0001). Notably, one study (Liu et al., 2019) showed that positive thinking intervention also had a significant improvement effect on depression in thyroid cancer patients (*p* = 0.0009).

The results of the subgroup analyses clearly indicate that positive thinking interventions have a positive impact on anxiety, depression and quality of life in patients with malignant tumors. However, the heterogeneity that existed across studies, particularly in quality of life assessment, suggested that the choice of assessment tool may be a key influencing factor.

Firstly, there were significant differences in the performance of different assessment tools in the assessment of quality of life. The FACT scale showed a high degree of heterogeneity due to its inclusion of multiple specificity modules. This may be due to the fact that these modules are designed for different health dimensions, resulting in greater variability in subjects’ quality of life scores across studies (Webster et al., 2003). In contrast, the QLQ-C30 and SF-36 scales showed lower heterogeneity in assessing quality of life. The QLQ-C30 scale is designed for cancer patients and has a uniform structure, thus providing more consistent assessment results across studies (Aaronson et al., 1993), while the SF-36 scale is effective in reducing heterogeneity due to its broad generalisability, especially in cross-cultural studies (Ware and Sherbourne, 1992). These findings suggest that the choice of assessment tool not only directly affects the reliability and consistency of the findings, but may also determine the heterogeneity of the findings to some extent.

In addition, there were differences in the effects of different types of positive thinking interventions on the psychological state and quality of life of patients with malignant tumors. Positive thought cognitive therapy showed consistent and significant effects in the alleviation of anxiety and depression with low heterogeneity, suggesting high consistency in the effects of this intervention across studies. In contrast, the effects of Positive Mindfulness Stress Reduction Therapy in the alleviation of anxiety and depressed mood showed a high degree of heterogeneity, which may be attributed to the fact that the method of implementation of this therapy and the characteristics of the subjects varied considerably across studies, leading to a low degree of reproducibility of the findings. Similarly, positive thinking meditation showed significant effects in the relief of anxiety and depression, but the number of relevant studies is limited and more evidence is needed to support this.

Tumor type is also an important factor influencing the effectiveness of positive thinking interventions. Studies have shown (Lengacher et al., 2021) that breast cancer patients showed significant relief of anxiety and depression and improved quality of life after receiving positive thinking intervention, which may be due to the better responsiveness of breast cancer patients to positive thinking intervention in terms of emotional coping strategies. In contrast, positive thinking intervention had a more limited effect on improving psychological status and quality of life in nasopharyngeal and rectal cancer patients, suggesting that patients with different types of tumors may require more individualized intervention programs. In addition, there were significant differences in the improvement effects of psychological status and quality of life between patients with gynecological tumors and thyroid cancer after positive thinking intervention, which further suggests the important role of tumor type in positive thinking intervention.

### 3.6 Publication bias

**Figure 7:**
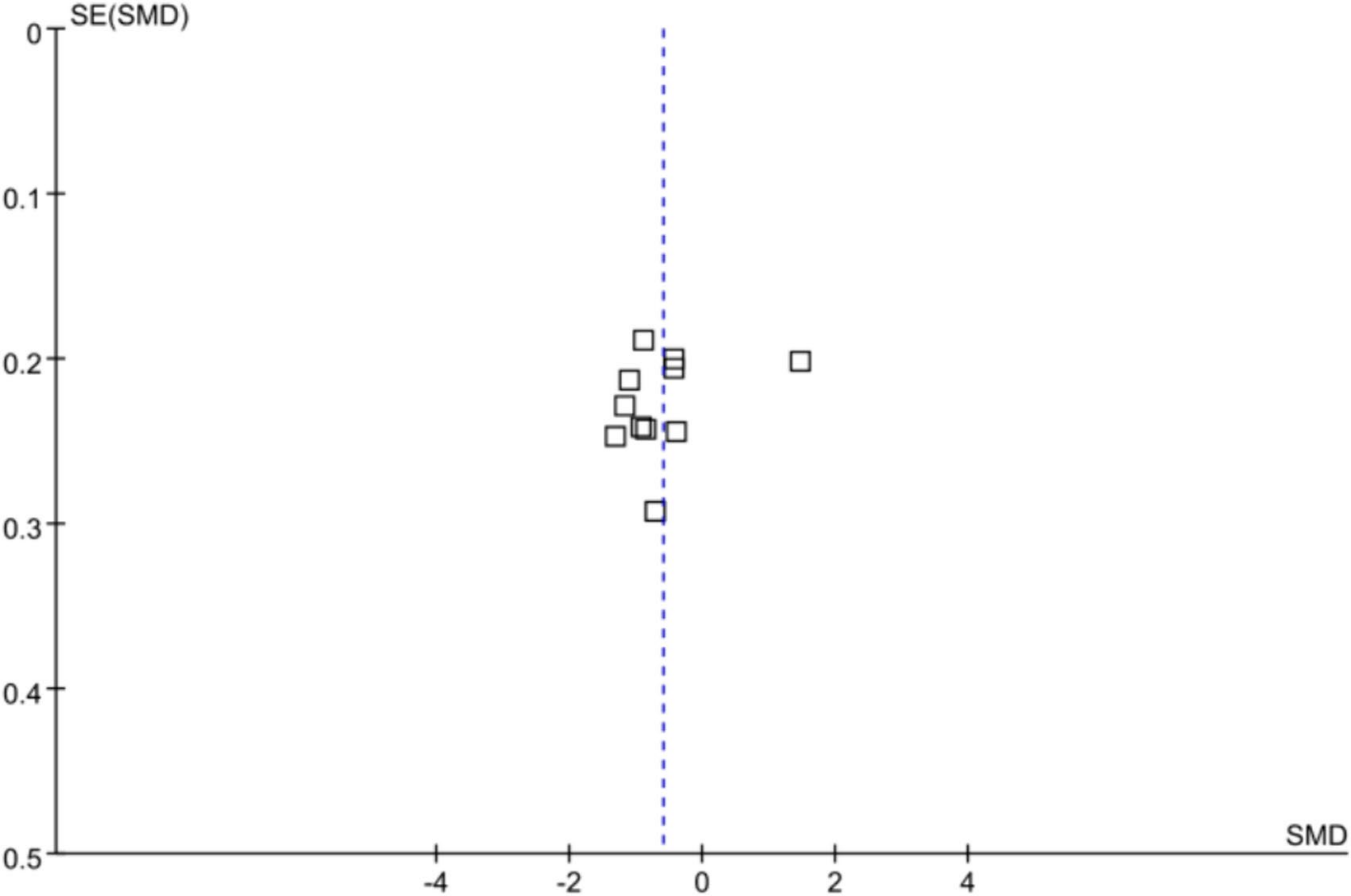
Funnel plot.

In this study, anxiety was used as the outcome measure to analyze publication bias. The results indicated a relatively even distribution of included studies, with only a few studies scattered, possibly due to the heterogeneity among studies. This suggests the presence of mild publication bias in the included studies, as detailed in Figure 7.

## 4 Discussion

### 4.1 Mindfulness intervention effectively alleviates negative emotions such as anxiety and depression in patients with malignant tumor

McCloy et al. (2022) and Lin et al. (2022) indicate that mindfulness intervention effectively alleviates anxiety and depression in cancer patients, which is consistent with the findings of this study. In psychological research, mindfulness practices have been found to help patients enhance acceptance and awareness, make changes within their capabilities after gaining clear awareness, cultivate stable mental states, and improve emotional regulation, thereby achieving the effect of alleviating anxiety and depression (Segal et al., 2002). Additionally, in psychosomatic medicine, mindfulness interventions can improve negative emotions such as anxiety and depression by influencing the central nervous system and immune system (Davidson et al., 2003). Firstly, in the central nervous system: mindfulness interventions can regulate brain regions related to emotion control by influencing the plasticity of the central nervous system, increasing activity in the prefrontal cortex, and reducing the volume of the right amygdala, thereby helping patients alleviate anxiety and depression (Gotink et al., 2018). Imaging studies have shown that mindfulness practices can alter brain structure and function, especially in areas related to emotional processing and attention regulation, as observed through functional magnetic resonance imaging (fMRI) (Hölzel et al., 2011). Secondly, in the immune system: mindfulness training can enhance immune system function, including increasing salivary immunoglobulins and immune cells, reducing inflammatory cytokines (such as IL-6 and TNFα), and C-reactive protein, and other emotion-regulating biomarkers, thus alleviating anxiety, depression, and other negative emotions (Creswell et al., 2016; Liu et al., 2024). These findings provide strong theoretical support for mindfulness interventions to help patients better manage emotions and cope with stress. Li et al. (2023) found that an 8-week mindfulness intervention period showed better results in alleviating negative emotions, which aligns with the design of standard Mindfulness-Based Stress Reduction (MBSR) courses. This highlights the importance of the 8-week duration in promoting mental health. However, individual differences affect the demand for intervention duration, suggesting that a more personalized approach may be more effective in mindfulness interventions. Further research suggests (Carlson, 2019) that considering the complexity of emotional and psychological needs in malignant tumor patients, intervention design should be flexible to adapt to the specific needs of different individuals. Moreover, exploring new intervention formats, such as adjusting the frequency and intensity of practices or customizing content tailored to emotional and physiological needs, may provide more targeted support to patients (Garland et al., 2019). With advancements in technology and the development of tools for digital intervention, Digital Mindfulness Interventions (DMIs) offer a new possibility, making interventions more flexible and personalized (Fisher et al., 2020). Through the use of mobile applications or online platforms, patients can engage in mindfulness practices autonomously according to their own schedules and needs, which is particularly beneficial for patients with limited time or physical conditions. Future research needs to explore the effectiveness of personalized interventions, especially in malignant tumor patients, considering different intervention durations, intensities, and formats, as well as how to improve the accessibility and engagement of interventions through technological means.

### 4.2 Mindfulness intervention can enhance the quality of life for malignant tumor patients

Mindfulness intervention, comprising mindfulness meditation, breathing exercises, yoga, body scans, and others, aims to cultivate individuals’ ability to monitor attention and acceptance, thereby enhancing emotional regulation (Segal et al., 2002). This study found that mindfulness intervention effectively improves the quality of life of malignant tumor patients, which aligns with the findings of Wang et al. (2022). Its comprehensive effect is evident in the significant reduction of depressive and anxiety symptoms. The amelioration of these psychological symptoms directly correlates with patients’ overall well-being and quality of life. However, the study by Zhu and Zhou (2022) suggested that mindfulness intervention may not effectively enhance the quality of life for patients with inflame quality of life matory bowel disease (IBD), possibly due to individual variances and disease-specific factors. Mindfulness intervention aids patients in engaging more effectively in daily activities and social interactions by alleviating negative emotions, thereby improving their quality of life. Mindfulness fosters awareness of the mind–body connection, assisting patients in better understanding their bodily signals and psychologically preparing for the treatment process (Piet et al., 2012). Mindfulness training holds promise for enduring effects, as patients can continue independent practice post formal training to sustain its benefits (Lengacher et al., 2012). Mindfulness serves as a non-pharmacological treatment modality to complement the care of malignant tumor patients. Further research may need to explore the specific mindfulness activities most effective in enhancing the quality of life and how to integrate mindfulness practices with traditional treatments to optimize their impact on patient well-being. Additionally, considering the potential lasting effects of mindfulness training, researchers should evaluate the long-term effects of patients’ continued independent practice post formal training.

### 4.3 Limitations

This study has the following limitations: (1) Limitations in language and literature databases: This study only includes Chinese and English databases, potentially overlooking important research findings in other languages. This may restrict the representativeness of the research results. (2) Limitations in study duration: The study primarily focuses on the short-term effects (8 weeks) of mindfulness intervention, lacking evaluation of long-term effects (beyond 8 weeks). This may hinder a comprehensive understanding of the long-term benefits of mindfulness intervention. (3) Diversity in intervention measures: The study encompasses various forms of mindfulness intervention, differing in intervention duration, frequency, and follow-up time. Such diversity may contribute to result heterogeneity, complicating meta-analysis (4) Lack of consideration for cancer patient classification: The study does not account for the types, stages, or other characteristics of malignant tumors, potentially overlooking differences in response to mindfulness intervention among patients with different tumor types. (5) Inconsistency in assessment tools and sample size issues: The utilization of diverse assessment tools may hinder comparisons and result interpretation. Moreover, small sample sizes in some studies may limit the effectiveness of statistical pooling and increase result heterogeneity.

## 5 Conclusion

In summary, positive thinking interventions have a significant positive impact on anxiety, depression and quality of life in patients with malignant tumors. However, differences in assessment tools, choice of intervention, and tumor type largely determined the heterogeneity of the findings. In future studies, it is crucial to address these influencing factors in depth. By optimizing the design of positive thinking interventions and providing more precise and personalized psychological support, the needs of patients with different types of tumors can be better served.

## Data Availability

The original contributions presented in the study are included in the article/Supplementary material, further inquiries can be directed to the corresponding author.

## References

[ref1] Aaronson N. K. Ahmedzai S. Bergman B. Bullinger M. Cull A. Duez N. J. . (1993). The European Organization for Research and Treatment of Cancer QLQ-C30: a quality-of-life instrument for use in international clinical trials in oncology. J. Natl. Cancer Inst. 85, 365–376. doi: 10.1093/jnci/85.5.365, PMID: 8433390

[ref2] Bower J. E. Irwin M. R. (2016). Mind-body therapies and control of inflammatory biology: a descriptive review. Brain Behav. Immun. 51, 1–11. doi: 10.1016/j.bbi.2015.06.012, PMID: 26116436 PMC4679419

[ref3] Cao M. M. Chen W. Q. (2019). Epidemiology of cancer in China and the current status of prevention and control. Chin. J. Clin. Oncol. 46, 145–149. doi: 10.3969/j.issn.1000-8179.2019.03.283

[ref4] Carlson L. E. (2019). Mindfulness-based interventions for physical conditions: a narrative review evaluating levels of evidence. ISRN Psychiatry 2019:1238017. doi: 10.1155/2019/1238017 PMC3671698 23762768

[ref5] Chen Y. Y. (2021). Application of rational emotive behavior therapy combined with mindfulness meditation training in young patients with breast cancer. Hengyang, China: University of South China.

[ref6] Creswell J. D. Irwin M. R. Burklund L. J. Lieberman M. D. Arevalo J. M. G. Ma J. . (2016). Mindfulness-based stress reduction training reduces loneliness and pro-inflammatory gene expression in older adults: a small randomized controlled trial. Brain Behav. Immun. 26, 1095–1101. doi: 10.1016/j.bbi.2012.07.006 PMC3635809 22820409

[ref7] Davidson R. J. Kabat-Zinn J. Schumacher J. Rosenkranz M. Muller D. Santorelli S. F. . (2003). Alterations in brain and immune function produced by mindfulness meditation. Psychosom. Med. 65, 564–570. doi: 10.1097/01.PSY.0000077505.67574.E3 12883106

[ref8] Ferlay J. Ervik M. Lam F. Colombet M. Mery L. Piñeros M. . (2020). Global cancer observatory: cancer today. Lyon, France: International Agency for Research on Cancer.

[ref9] Fisher N. Mead B. R. Lattie E. Mohr D. C. (2020). Integrating technology into standard mindfulness-based interventions: bridging the gap between research and practice. J. Cogn. Psychother. 34, 291–304. doi: 10.1891/JCPSY-D-19-00039

[ref10] Garland E. L. Farb N. A. Goldin P. Fredrickson B. L. (2019). Mindfulness broadens awareness and builds eudaimonic meaning: a process model of mindful positive emotion regulation. Psychol. Inq. 30, 128–152. doi: 10.1080/1047840X.2019.1654810 PMC4826727 27087765

[ref11] Garland E. L. Zhou Y. Gonzalez J. A. Rodriguez M. Nakamura Y. Campbell-Sills L. . (2019). Mindfulness-oriented recovery enhancement improves stress biomarkers and self-reported health in cancer survivors: a randomized controlled trial. J. Behav. Med. 42, 556–569. doi: 10.1007/s10865-018-9990-1

[ref12] Gotink R. A. Vernooij M. W. Ikram M. A. Niessen W. J. Krestin G. P. Hofman A. . (2018). Meditation and yoga practice are associated with smaller right amygdala volume: the rotter-dam study. Brain Imaging Behav. 12, 1631–1639. doi: 10.1007/s11682-018-9826-z, PMID: 29417491 PMC6302143

[ref13] Haller H. Winkler M. M. Klose P. Dobos G. Kümmel S. Cramer H. (2017). Mindfulness-based interventions for women with breast cancer: an updated systematic review and meta-analysis. Acta Oncol. 56, 1665–1676. doi: 10.1080/0284186X.2017.1342862, PMID: 28686520

[ref14] Hao M. Tan M. Y. Wu Q. Wang J.X. Zhang X. Y. Li H. . (2019). Effect of group mindfulness cognitive therapy on the depression, anxiety and quality of life in patients with breast cancer during chemotherapy. J. Chengdu Med. Coll. 14, 485–489.

[ref15] Hölzel B. K. Carmody J. Vangel M. Congleton C. Yerramsetti S. M. Gard T. . (2011). Mindfulness practice leads to increases in regional brain gray matter density. Psychiatry Res. Neuroimaging 191, 36–43. doi: 10.1016/j.pscychresns.2010.08.006, PMID: 21071182 PMC3004979

[ref16] Johns S. A. Brown L. F. Beck-Coon K. Monahan P. O. Tong Y. Kroenke K. (2015). Randomized controlled pilot study of mindfulness-based stress reduction for persistently fatigued cancer survivors. Psychooncology 24, 885–893. doi: 10.1002/pon.3648, PMID: 25132206 PMC4331267

[ref17] Lengacher C. A. Kip K. E. Barta M. Post-White J. Jacobsen P. B. Groer M. . (2012). A pilot study evaluating the effect of mindfulness-based stress reduction on psychological status, physical status, salivary cortisol, and Interleukin-6 among advanced-stage cancer patients and their caregivers. J. Holist. Nurs. 30, 170–185. doi: 10.1177/0898010111435949, PMID: 22442202

[ref18] Lengacher C. A. Shelton M. M. Reich R. R. Barta M. K. Johnson-Mallard V. Moscoso M. S. . (2021). Mindfulness-based stress reduction (MBSR) in post-treatment breast cancer patients: an examination of symptoms and symptom clusters. J. Behav. Med. 44, 297–311. doi: 10.1007/s10865-020-00173-4 21506018

[ref19] Li J. Jiang X. Y. Hua Y. (2019). The application of mindfulness-based stress reduction in patients with gynecologic malignancies. J. Qilu Nurs. 25, 64–67. doi: 10.3969/j.issn.1006-7256.2019.14.025

[ref20] Li J. Li C. Puts M. Wu Y. C. Lyu M. M. Yuan B. . (2023). Effectiveness of mindfulness-based interventions on anxiety, depression, and fatigue in people with lung cancer: a systematic review and meta-analysis. Int. J. Nurs. Stud. 140:104447. doi: 10.1016/j.ijnurstu.2023.104447, PMID: 36796118

[ref21] Li G. H. Zhang G. C. Zhu S. J. (2021). Effect of mindfulness decompression training for mental state and quality of life in patients with nasopharyngeal carcinoma. Int. J. Nurs. 40, 2770–2774.

[ref22] Lin L. Y. Lin L. H. Tzeng G. L. Huang Y. H. Tai J. F. Chen Y. L. . (2022). Effects of mindfulness-based therapy for cancer patients: a systematic review and meta-analysis. J. Clin. Psychol. Med. Settings 29, 432–445. doi: 10.1007/s10880-022-09862-z, PMID: 35249176

[ref23] Liu S. J. Liu B. J. Li G. Chen Y. Q. Zhang M. Huang L. T. . (2024). Meta-analysis of the impact of mindfulness training on athletes’ performance-related metrics. Chin. Ment. Health J. 38, 368-376+2. doi: 10.3969/j.issn.1000-6729.2024.04.014

[ref24] Liu T. Zhang W. Xiao S. Xu L. Wen Q. Bai L. . (2019). Mindfulness-based stress reduction in patients with differentiated thyroid cancer receiving radioactive iodine therapy: a randomized controlled trial. Cancer Manag. Res. 11, 467–474. doi: 10.2147/CMAR.S183299 30655698 PMC6324610

[ref25] Lv W. P. (2016). Assessment of nursing intervention effects on improving quality of life in patients with malignant tumors after radiotherapy. Chinese Nurs. Manag. 16, 92–94.

[ref26] McCloy K. Hughes C. Dunwoody L. Marley J. Gracey J. (2022). Effects of mindfulness-based interventions on fatigue and psychological wellbeing in women with cancer: a systematic review and meta-analysis of randomised control trials. Psychooncology 31, 1821–1834. doi: 10.1002/pon.6046, PMID: 36221152 PMC9828570

[ref27] McCombie A. Jordan J. Mulder R. Dee K. Ong E. L. Zimmermann F. F. . (2023). A randomized controlled trial of mindfulness in recovery from colorectal cancer. Chin. J. Integr. Med. 29, 590–599. doi: 10.1007/s11655-023-3632-1, PMID: 36941505 PMC10027425

[ref28] Oberoi S. Yang J. Woodgate R. L. Niraula S. Banerji S. Israels S. J. . (2020). Association of mindfulness-based interventions with anxiety severity in adults with cancer: a systematic review and meta-analysis. JAMA Netw. Open 3:e2012598. doi: 10.1001/jamanetworkopen.2020.12598, PMID: 32766801 PMC7414391

[ref29] Pan S. Q. Lv Q. Qiu A. P. (2022). The effects of mindfulness-based stress reduction on pain, anxiety, depression, and quality of life in patients with nasopharyngeal carcinoma during radiotherapy. J. Navy Med. 43, 222–225. doi: 10.3969/j.issn.1009-0754.2022.02.026

[ref30] Park S. Sato Y. Takita Y. Tamura N. Ninomiya A. Kosugi T. . (2020). Mindfulness-based cognitive therapy for psychological distress, fear of cancer recurrence, fatigue, spiritual well-being, and quality of life in patients with breast cancer-a randomized controlled trial. J. Pain Symptom Manag. 60, 381–389. doi: 10.1016/j.jpainsymman.2020.02.017, PMID: 32105790

[ref31] Piet J. Würtzen H. Zachariae R. (2012). The effect of mindfulness-based therapy on symptoms of anxiety and depression in adult cancer patients and survivors: a systematic review and meta-analysis. J. Consult. Clin. Psychol. 80, 1007–1020. doi: 10.1037/a0028329, PMID: 22563637

[ref32] Qing Y. F. Gui D. Q. (2018). Application of mindfulness-based stress reduction in cervical cancer patients with chemoradiotherapy. Chinese J. Modern Nurs. 24, 1929–1933.

[ref33] Segal Z. V. Williams J. M. G. Teasdale J. D. (2002). Mindfulness-based cognitive therapy for depression: a new approach to preventing relapse. New York, NY: Guilford Press.

[ref34] Wang H. Y. Ye J. R. Xiao A. X. Li Z. Y. Chen Y. L. Zhang Q. Y. . (2022). Effect of mindfulness-based cancer recovery on mood, stress level, and quality of life in cancer patients: a meta-analysis. Chinese Evid. Based Nurs. 8, 36–41. doi: 10.12102/j.issn.2095-8668.2022.01.007

[ref35] Ware J. E. Jr. Sherbourne C. D. (1992). The MOS 36-item short-form health survey (SF-36): I. Conceptual framework and item selection. Med. Care 30, 473–483. doi: 10.1097/00005650-199206000-00002 1593914

[ref36] Webster K. Cella D. Yost K. (2003). The functional assessment of chronic illness therapy (FACIT) measurement system: properties, applications, and interpretation. Health Qual. Life Outcomes 1:79. doi: 10.1186/1477-7525-1-79, PMID: 14678568 PMC317391

[ref37] Zhang R. L. Cheng X. Q. Yang Y. Z. (2018). Effects of aerobic exercises combined with mindfulness-based stress reduction on postoperative breast cancer patients. J. Nurs. Sci. 33, 76–78+89. doi: 10.3870/j.issn.1001-4152.2018.18.076

[ref38] Zhu P. Liu X. Shang X. Chen Y. Chen C. Wu Q. (2023). Mindfulness-based stress reduction for quality of life, psychological distress, and cognitive emotion regulation strategies in patients with breast cancer under early chemotherapy-a randomized controlled trial. Holist Nurs. Pract. 37, 131–142. doi: 10.1097/HNP.0000000000000580 37070838

[ref39] Zhu T. R. Zhou Y. X. (2022). The effect of mindfulness-based intervention on anxiety, depression and quality of life in patients with inflammatory bowel disease: a meta-analysis. Nurs. J. Chinese People’s Liberation Army 39:61–64+80. doi: 10.3969/j.issn.1008-9993.2022.04.016

