# Supplementary material for: The impact of mindfulness intervention on negative emotions and quality of life in malignant tumor patients: a systematic review and meta-analysis
Source: Front Psychol. 2024 Sep 18;15:1443516. doi: 10.3389/fpsyg.2024.1443516 (PMC11445068; doi:10.3389/fpsyg.2024.1443516)
Supplement: Supplementary file 2 [file Data_Sheet_2.PDF]

PUBMED

("Mindfulness" OR "mindfulness meditation" OR "insight meditation" OR "mindfulness based stress reduction" OR "mindfulness based cognitive therapy" OR "MBSR") AND ("Malignant tumor" OR "malignancies" OR "malignant tumour" OR "Malignant neoplasm" OR "malignant tumors" OR "malignant cancer" OR "Malignant neoplasms" OR "malignant carcinoma" OR "cancer") AND ("quality of life" OR "Living quality" OR "The Quality of Life" OR "Quality-of-life" OR "Life-quality" OR "quality of lives" OR "Quality of living" OR "Life quality") AND ("anxiety" OR "Anxiety" OR "Anxiety and depression")

Embase

('mindfulness'/exp OR mindfulness OR 'mindfulness meditation'/exp OR 'mindfulness meditation' OR (('mindfulness'/exp OR mindfulness) AND ('meditation'/exp OR meditation)) OR 'insight meditation' OR (('insight'/exp OR insight) AND ('meditation'/exp OR meditation)) OR 'mindfulness based stress reduction'/exp OR 'mindfulness based stress reduction' OR (('mindfulness'/exp OR mindfulness) AND based AND ('stress'/exp OR stress) AND ('reduction'/exp OR reduction)) OR 'mindfulness based cognitive therapy'/exp OR 'mindfulness based cognitive therapy' OR (('mindfulness'/exp OR mindfulness) AND based AND cognitive AND ('therapy'/exp OR therapy)) OR mbsr) AND ('malignant tumor':ab,ti OR malignancies:ab,ti OR 'malignant tumour':ab,ti OR 'malignant neoplasm':ab,ti OR 'malignant tumors':ab,ti OR 'malignant cancer':ab,ti OR 'malignant neoplasms':ab,ti OR 'malignant carcinoma':ab,ti OR cancer:ab,ti) AND ('living quality':ab,ti OR 'the quality of life':ab,ti OR 'quality of life':ab,ti OR 'quality of lives':ab,ti OR 'quality of living':ab,ti OR 'life quality':ab,ti) AND (anxiety:ab,ti OR 'or anxiety':ab,ti) AND anxiety:ab,ti AND depression:ab,ti

Web of Science

Mindfulness OR mindfulness meditation OR insight meditation OR mindfulness based stress reduction OR mindfulness based cognitive therapy OR MBSR (Topic) and Malignant tumor or malignancies or malignant tumour or Malignant neoplasm or malignant tumors or malignant cancer or Malignant neoplasms or malignant carcinoma or malignant tumours or cancer (Topic) and quality of life OR Living quality OR The Quality of Life OR Quality-of-life OR Life-quality OR quality of lives OR Quality of living OR Life quality (Topic) and anxiety or anguish angst or Anxiety and Anxiety and depression (Topic)

Cochrane Library

(Mindfulness OR mindfulness meditation OR insight meditation OR mindfulness based stress reduction OR mindfulness based cognitive therapy OR MBSR)and (Malignant tumor or malignancies or malignant tumour or Malignant neoplasm or malignant tumors or malignant cancer or Malignant neoplasms or malignant carcinoma or malignant tumours or cancer)and(quality of life OR Living quality OR The Quality of Life OR Quality-of-life OR Life-quality OR quality of lives OR Quality of living OR Life quality)and (anxiety or anguish Anqst or Anxiety and Anxiety and depression)and(arandomized controlled trial OR randomized controlled trials OR randomized clinical trial OR randomized control trial OR randomized controlled study OR randomized control trials OR randomized clinical trials OR randomized

controlled trails OR randomized controlled trail OR randomised controlled trials)

知网

SU%=('正念'+'正念冥想'+'正念疗法'+'正念减压'+'正念训练'+'正念干预'+'正念认知疗法'+'正念练习')*('恶性肿瘤'+'癌症'+'癌'+'肿瘤')*('生活质量'+'生存质量'+'生命质量')*('焦虑'+'抑郁'+'负面情绪'+'负性情绪')

维普

M=（正念+正念冥想+正念疗法+正念减压+正念训练+正念干预+正念认知疗法+正念练习）AND M=（恶性肿瘤+癌症+肿瘤+癌）AND M=（生活质量+生存质量+生命质量）AND M=（焦虑+抑郁+负面情绪+负性情绪）

万方

题名或关键词:(正念 or 正念冥想 or 正念疗法 or 正念减压 or 正念训练 or 正念干预 or 正念认知疗法 or 正念练习) and 题名或关键词:(恶性肿瘤 or 癌症 or 肿瘤 or 癌) and 题名或关键词:(生活质量 or 生存质量 or 生命质量) and 题名或关键词:(焦虑 or 抑郁 or 负面情绪 or 负性情绪)

中国生物医学文献数据库

("正念"[常用字段:智能] OR "正念冥想"[常用字段:智能] OR "正念疗法"[常用字段:智能] OR "正念减压"[常用字段:智能] OR "正念训练"[常用字段:智能] OR "正念干预"[常用字段:智能] OR "正念认知疗法"[常用字段:智能] OR "正念练习"[常用字段:智能]) AND ("癌"[常用字段:智能] OR "癌症"[常用字段:智能] OR "恶性肿瘤"[常用字段:智能] OR "肿瘤"[常用字段:智能]) AND ("生活质量"[常用字段:智能] OR "生存质量"[常用字段:智能] OR "生命质量"[常用字段:智能]) AND ("焦虑"[常用字段:智能] OR "抑郁"[常用字段:智能] OR "负面情绪"[常用字段:智能] OR "负性情绪"[常用字段:智能])
