# Supplementary figures and images for: The impact of mindfulness intervention on negative emotions and quality of life in malignant tumor patients: a systematic review and meta-analysis
Source: Front Psychol. 2024 Sep 18;15:1443516. doi: 10.3389/fpsyg.2024.1443516 (PMC11445068; doi:10.3389/fpsyg.2024.1443516)

# PRISMA flow diagram

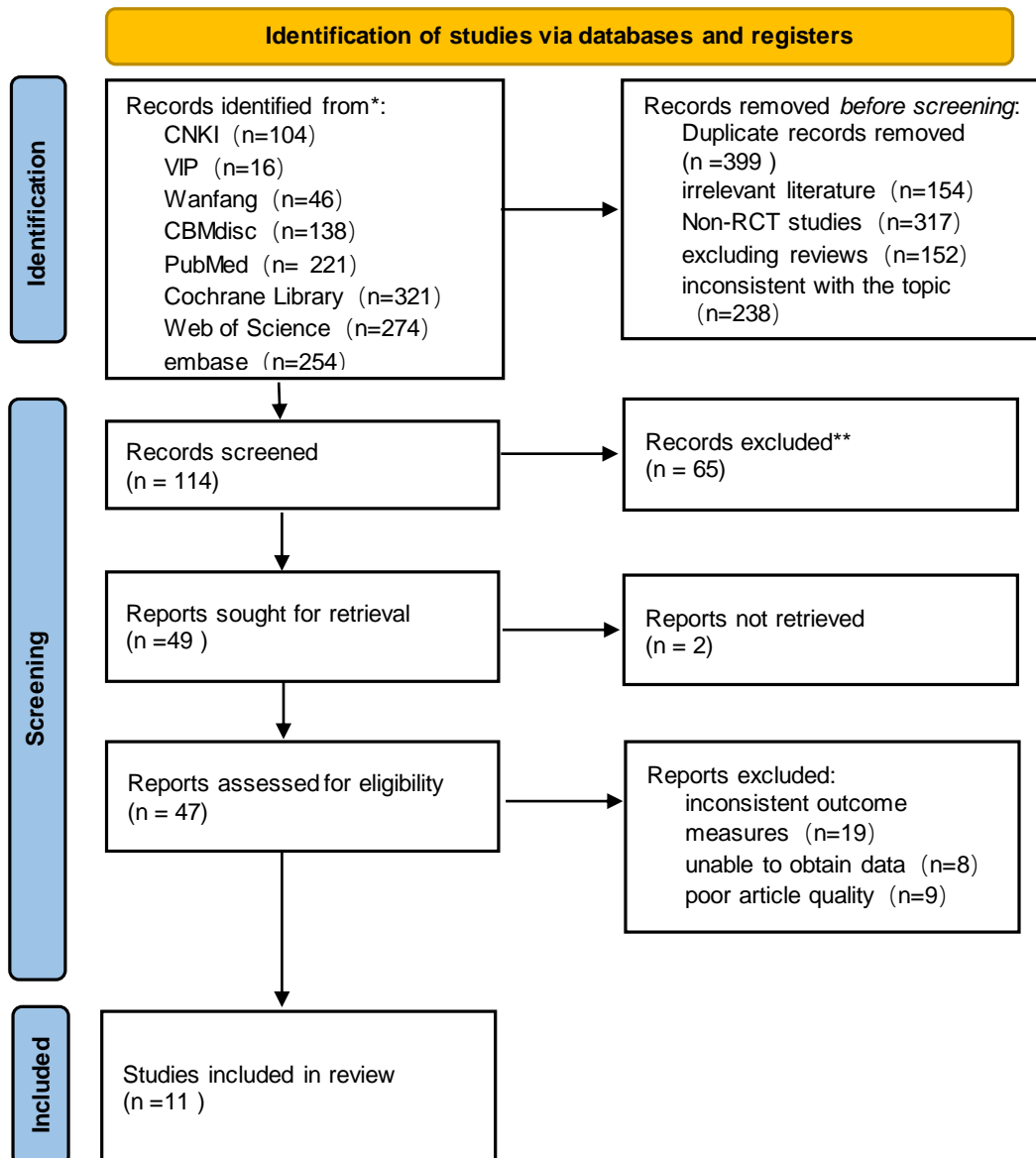

Supplement: Supplementary file 3 [file Data_Sheet_3.PDF]
