# Supplementary material for: The impact of mindfulness intervention on negative emotions and quality of life in malignant tumor patients: a systematic review and meta-analysis
Source: Front Psychol. 2024 Sep 18;15:1443516. doi: 10.3389/fpsyg.2024.1443516 (PMC11445068; doi:10.3389/fpsyg.2024.1443516)
Supplement: Supplementary file 4 [file Data_Sheet_4.PDF]

Included Studies	Publication Date	Type of Tumor	Sample Size Control Group/Observation Group	Intervention Measures		Implementation personnel	Duration of Intervention	Evaluation time	Outcome Measures	Assessment Tools
				Control Group	Observation Group					
Zhang RL[9]	2018	lymphoma	45/45	Usual Care	Usual Care+Mindfulness-Based Stress Reduction Combined with Aerobic Exercise Training	Psychologists	6 weeks, 6 times/week 1 time/60 minutes	Baseline, 6 weekends after intervention	Anxiety、Depression、Quality of Life、Pressure、Subjective Well-being、Expectation Level	SAS、SDS、SF-36、CPSS、MUNSH
Chen YY[10]	2021	lymphoma	38/38	Usual Care	Usual Care+Rational Emotive Behavior Therapy Combined with Mindfulness Meditation	Doctors, nurses, and psychological counselors	8 weeks, 2 times/week 1 time/40 minutes	Baseline, 8 weekends after intervention	Anxiety、Depression、Quality of Life、Self-Acceptance	SAS、SDS、FACT-B、SAQ
Hao M[11]	2019	lymphoma	65/65	Usual Care	Usual Care+Group Mindfulness Cognitive Therapy	nurse	8 weeks, 2 times/week 1 time/1 hour	Baseline, 8 weekends after intervention	Anxiety、Depression、Quality of Life	SAS、SDS、QLQ-C30
Pan SQ[12]	2022	Nasopharyngeal Carcinoma NPC	25/25	Usual Care+psychological nursing	Usual Care+psychological nursing+Mindfulness-Based Stress Reduction	Professional trained nurses	8 weeks, 4-5 times/week 1 time/45 minutes	Baseline, 8 weekends after intervention	Anxiety、Depression、Quality of Life、pain	HADS、VAS、FACT-H &N
Li J[13]	2019	gynecologic malignancy	40/40	Usual Care	Usual Care+Mindfulness-Based Stress Reduction	Professional trained nurses	8 weeks, once a week 1 time/2~3h	Baseline, 8 weekends after intervention	Anxiety、Depression、Quality of Life、Pressure、Self-assessment of symptoms	SAS、SDS、SF-36、CPSS
Qing YF[14]	2018	cervix	48/48	Usual Care	Usual Care+Mindfulness-Based Stress Reduction	Professional trained nurses	8 weeks, once a week 1 time/3 hours	Baseline, 8 weekends after intervention	Anxiety、Depression、Quality of Life、Fatigue、sleep quality、	SAS、SDS、FACT-CX、PSQI
Li GH[15]	2021	Nasopharyngeal Carcinoma NPC	63/63	Usual Care	Usual Care+Mindfulness-Based Stress Reduction	Psychologists	8 weeks, once a week 1 time/30 minutes	Baseline, 8 weekends after intervention	Anxiety、Depression、Quality of Life、Pressure、Self-assessment of symptoms	BAI、BDI、QLQ-C30、CPSS
Park S.[16]	2020	lymphoma	36/38	Usual Care	Usual Care+Mindfulness Cognitive Therapy	Psychologists, psychiatrists, nurses	8 weeks, once a week 1 time/2 hours	Baseline, 8 weeks after intervention, and 12 weeks after intervention	Anxiety、Depression、Quality of Life、wearily、mental health、Fear of Cancer Recurrence	HADS、FACT-G、CARS、BFI、FACIT-Sp
Liu T.[17]	2019	thyroid cancer	53/49	Usual Care	Usual Care+Mindfulness-Based Stress Reduction	Psychologists	8 weeks, once a week	Baseline, 8 weekends after intervention, and at the end of 3 months after intervention	Anxiety、Depression、Quality of Life、wearily	SAS、SDS、QLQ-C30
Zhu P.[18]	2023	lymphoma	51/50	Usual Care	Mindfulness-Based Stress Reduction	Professional trainers	8 weeks, once a week 1 time/2 hours	Baseline, 8 weekends after intervention	Anxiety、Depression、Quality of Life、Cognitive Emotion	SAS、SDS、FACT-B
McCombie A.[19]	2023	rectum	33/35	Psychological Education+Cognitive Behavioral Skills and Support	Mindfulness intervention	psychologist	8 weeks, once a week 1 time/2 hours	Baseline, 8 weekends after intervention, and at the end of 6 months after intervention	Anxiety、Depression、Quality of Life	HADS、QOL

Notes：QLQ-C30，quality of life questionnaire-core 30；SF-36，the 36- item Short From Health Survey；FACT，Functional Assessment of Cancer Therapy；SAS，self-raing anxiety scale；SDS，self-rating depression scale；HADS，hospital anxiety and depression scale；BAI，Beck Anxiety Inventory；BDI，Beck's Depression Inventory；PSQI，Pittsburgh sleep quality index；VAS，visnal anal-ogne scale；CPSS，Chinese Version Per-ceived Stress Scale
