# Supplementary material for: The impact of mindfulness intervention on negative emotions and quality of life in malignant tumor patients: a systematic review and meta-analysis
Source: Front Psychol. 2024 Sep 18;15:1443516. doi: 10.3389/fpsyg.2024.1443516 (PMC11445068; doi:10.3389/fpsyg.2024.1443516)
Supplement: Supplementary file 5 [file Data_Sheet_5.PDF]

1.Anxiety subgroup analysis

Study or Subgroup	Experimental Mean	Experimental SD	Experimental Total	Control Mean	Control SD	Control Total	Weight	Mean Difference IV, Random, 95% CI
**4.1.1 SAS**								
Chen YY 2021	46.89	9.82	38	54.97	7.18	38	8.0%	-8.08 [-11.95, -4.21]
Hao M 2019	42.1	5.81	62	47.25	5.67	61	9.5%	-5.15 [-7.18, -3.12]
Li J 2019	42.76	3.27	40	47.01	3.28	40	9.8%	-4.25 [-5.69, -2.81]
Liu T. 2019	48.97	9.82	49	60.44	10.83	53	7.9%	-11.47 [-15.48, -7.46]
Qing YF 2018	43.27	10.08	48	47.66	10.33	48	7.9%	-4.39 [-8.47, -0.31]
Zhang RL 2018	43.78	3.34	45	48.22	4.16	45	9.8%	-4.44 [-6.00, -2.88]
Zhu P. 2023	44.5	11.92	50	48.85	9.26	51	7.8%	-4.35 [-8.52, -0.18]
**Subtotal (95% CI)**			**332**			**336**	**60.7%**	**-5.53 [-7.03, -4.04]**
Heterogeneity: Tau² = 2.05; Chi² = 14.19, df = 6 (P = 0.03); I² = 58%								
Test for overall effect: Z = 7.25 (P < 0.00001)								
**4.1.2 HADS**								
McCombie A. 2023	4.24	2.83	35	5.55	4	33	9.7%	-1.31 [-2.97, 0.35]
Pan SQ 2022	7.15	1.74	25	8.34	1.65	25	10.0%	-1.19 [-2.13, -0.25]
Park S. 2020	3.83	3.04	38	6.64	3.64	36	9.8%	-2.81 [-4.34, -1.28]
**Subtotal (95% CI)**			**98**			**94**	**29.5%**	**-1.67 [-2.65, -0.69]**
Heterogeneity: Tau² = 0.29; Chi² = 3.24, df = 2 (P = 0.20); I² = 38%								
Test for overall effect: Z = 3.33 (P = 0.0009)								
**4.1.3 BAI**								
Li GH 2021	22.78	4.75	63	16.46	3.61	63	9.8%	6.32 [4.85, 7.79]
**Subtotal (95% CI)**			**63**			**63**	**9.8%**	**6.32 [4.85, 7.79]**
Heterogeneity: Not applicable								
Test for overall effect: Z = 8.41 (P < 0.00001)								
**Total (95% CI)**			**493**			**493**	**100.0%**	**-3.48 [-5.88, -1.09]**
Heterogeneity: Tau² = 14.68; Chi² = 188.43, df = 10 (P < 0.00001); I² = 95%								
Test for overall effect: Z = 2.85 (P = 0.004)								
Test for subgroup differences: Chi² = 131.04, df = 2 (P < 0.00001), I² = 98.5%								

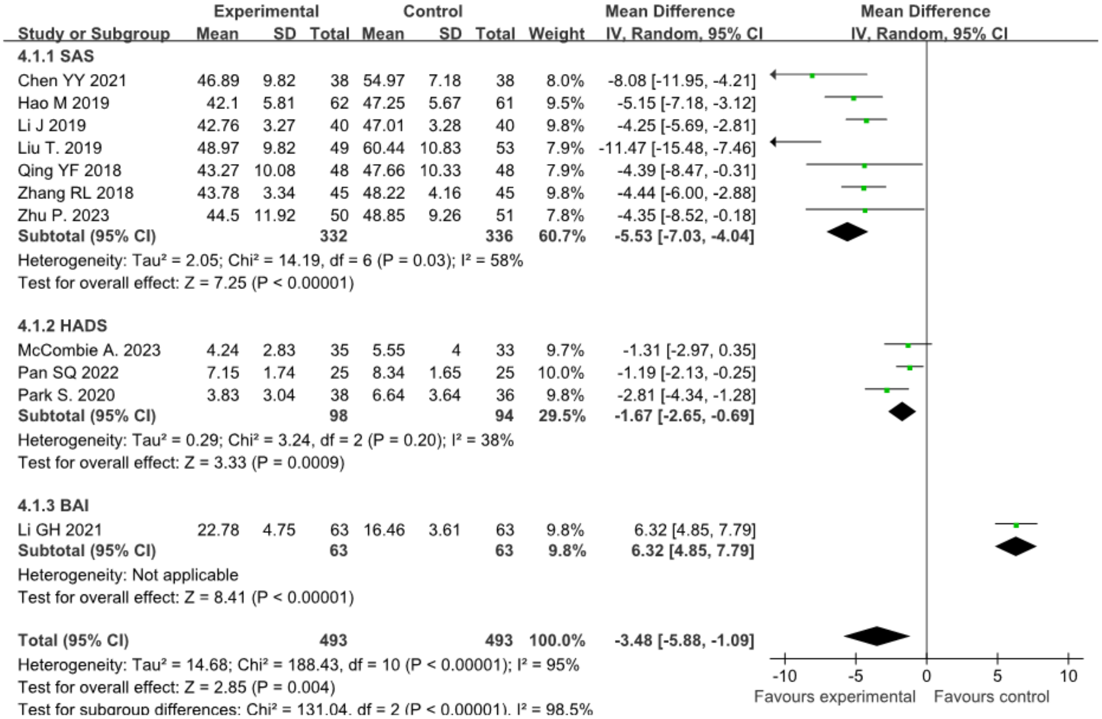

Subgroup analysis of assessment tools

Study or Subgroup	Experimental Mean	Experimental SD	Experimental Total	Control Mean	Control SD	Control Total	Weight	Std. Mean Difference IV, Random, 95% CI
**8.1.1 MBCT**								
Hao M 2019	42.1	5.81	62	47.25	5.67	61	9.3%	-0.89 [-1.26, -0.52]
Park S. 2020	3.83	3.04	38	6.64	3.64	36	9.0%	-0.83 [-1.31, -0.36]
**Subtotal (95% CI)**			**100**			**97**	**18.3%**	**-0.87 [-1.16, -0.58]**
Heterogeneity: Tau² = 0.00; Chi² = 0.04, df = 1 (P = 0.84); I² = 0%								
Test for overall effect: Z = 5.82 (P < 0.00001)								
**8.1.2 MBSR**								
Li GH 2021	22.78	4.75	63	16.46	3.61	63	9.3%	1.49 [1.09, 1.89]
Li J 2019	42.76	3.27	40	47.01	3.28	40	9.0%	-1.29 [-1.77, -0.80]
Liu T. 2019	48.97	9.82	49	60.44	10.83	53	9.2%	-1.10 [-1.52, -0.68]
McCombie A. 2023	4.24	2.83	35	5.55	4	33	9.0%	-0.38 [-0.86, 0.10]
Pan SQ 2022	7.15	1.74	25	8.34	1.65	25	8.7%	-0.69 [-1.26, -0.12]
Qing YF 2018	43.27	10.08	48	47.66	10.33	48	9.2%	-0.43 [-0.83, -0.02]
Zhang RL 2018	43.78	3.34	45	48.22	4.16	45	9.1%	-1.17 [-1.62, -0.72]
Zhu P. 2023	44.5	11.92	50	48.85	9.26	51	9.3%	-0.40 [-0.80, -0.01]
**Subtotal (95% CI)**			**355**			**358**	**72.7%**	**-0.49 [-1.15, 0.17]**
Heterogeneity: Tau² = 0.84; Chi² = 123.26, df = 7 (P < 0.00001); I² = 94%								
Test for overall effect: Z = 1.46 (P = 0.14)								
**8.1.3 Mindfulness Meditation**								
Chen YY 2021	46.89	9.82	38	54.97	7.18	38	9.0%	-0.93 [-1.40, -0.46]
**Subtotal (95% CI)**			**38**			**38**	**9.0%**	**-0.93 [-1.40, -0.46]**
Heterogeneity: Not applicable								
Test for overall effect: Z = 3.84 (P = 0.0001)								
**Total (95% CI)**			**493**			**493**	**100.0%**	**-0.60 [-1.08, -0.11]**
Heterogeneity: Tau² = 0.62; Chi² = 133.28, df = 10 (P < 0.00001); I² = 92%								
Test for overall effect: Z = 2.42 (P = 0.02)								
Test for subgroup differences: Chi² = 1.26, df = 2 (P = 0.53), I² = 0%								

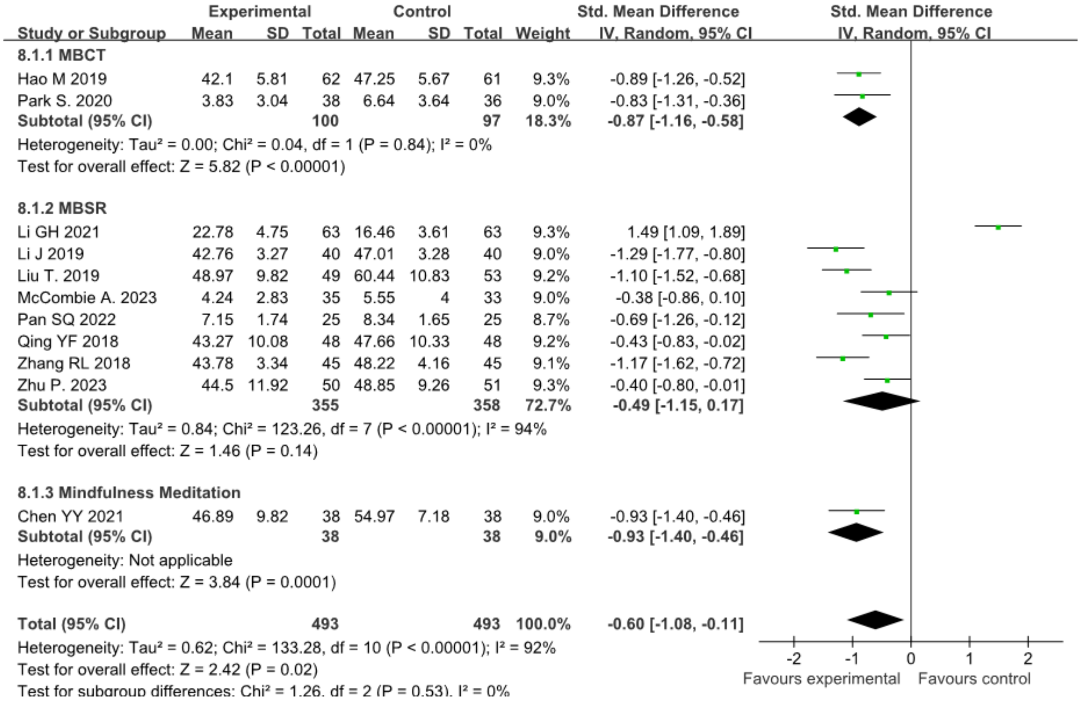

Positive thinking interventions

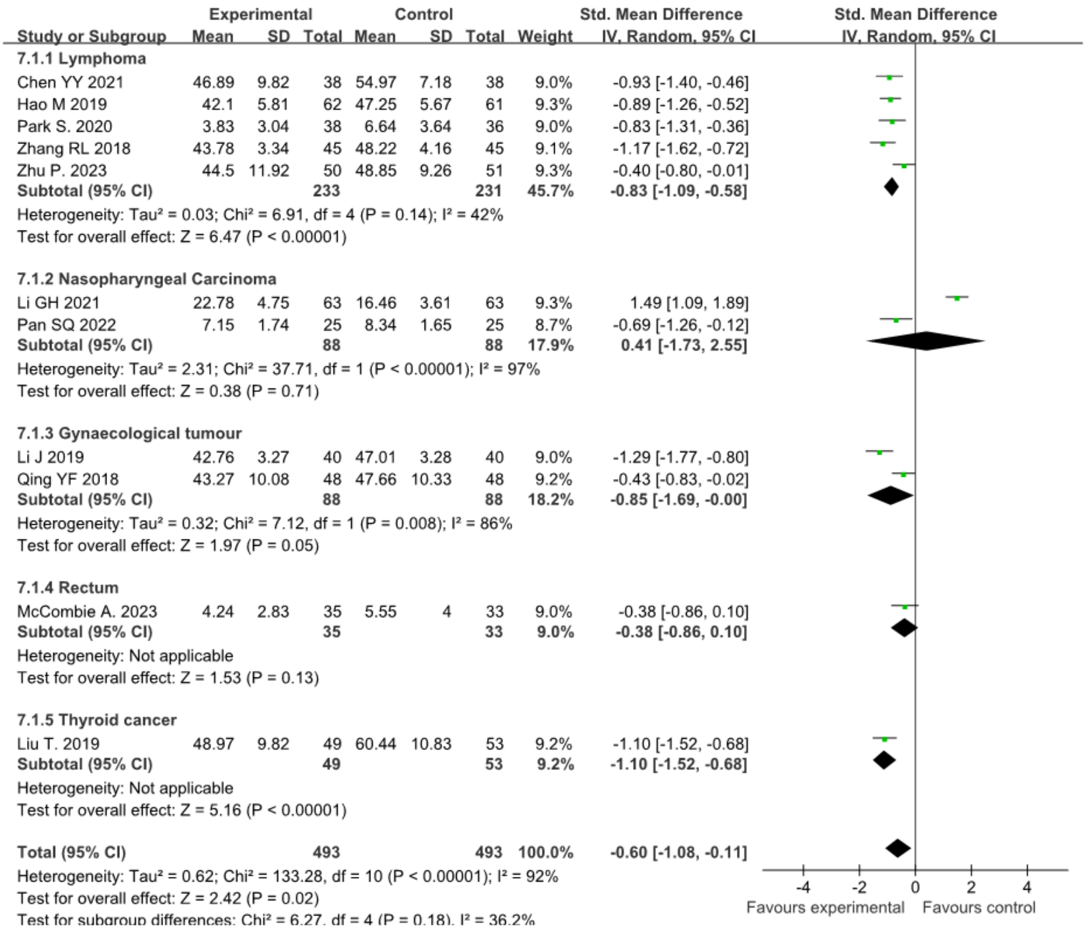

Tumour type

2. Subgroup analysis of depression

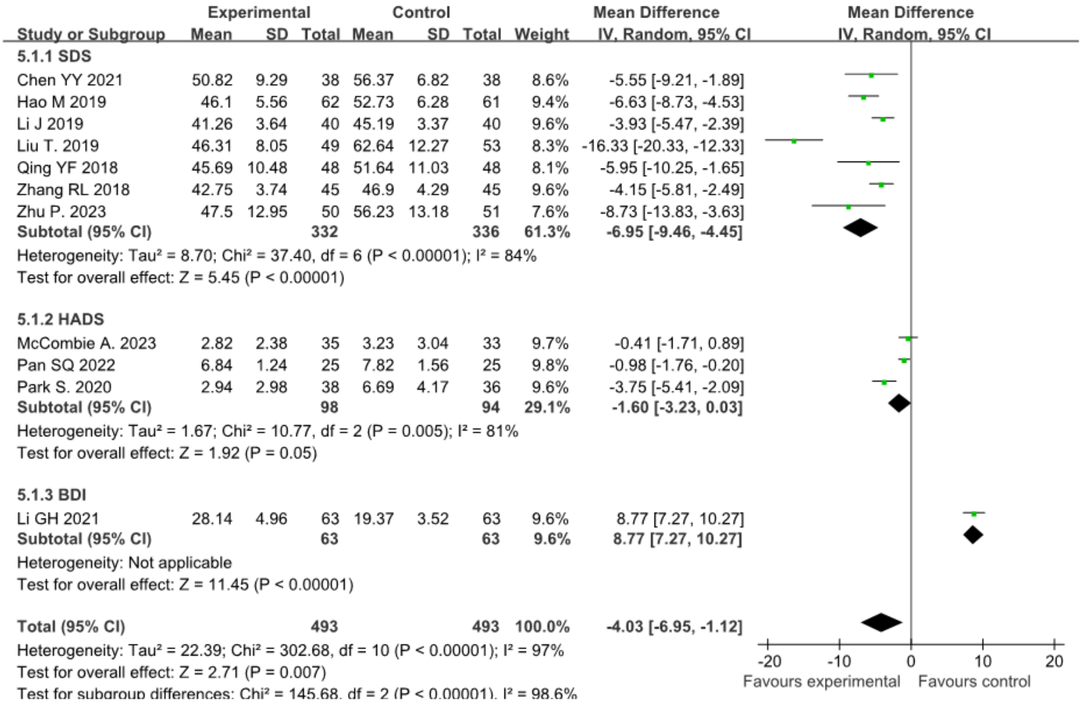

Subgroup analysis of assessment tools

Study or Subgroup	Experimental Mean	Experimental SD	Experimental Total	Control Mean	Control SD	Control Total	Weight	Std. Mean Difference IV, Random, 95% CI
**8.2.1 MBCT**								
Hao M 2019	46.1	5.56	62	52.73	6.28	61	9.2%	-1.11 [-1.49, -0.73]
Park S. 2020	2.94	2.98	38	6.69	4.17	36	9.0%	-1.03 [-1.52, -0.54]
**Subtotal (95% CI)**			**100**			**97**	**18.3%**	**-1.08 [-1.38, -0.78]**
Heterogeneity: Tau² = 0.00; Chi² = 0.07, df = 1 (P = 0.79); I² = 0%								
Test for overall effect: Z = 7.06 (P < 0.00001)								
**8.2.2 MBSR**								
Li GH 2021	28.14	4.96	63	19.37	3.52	63	9.1%	2.03 [1.59, 2.46]
Li J 2019	41.26	3.64	40	45.19	3.37	40	9.0%	-1.11 [-1.58, -0.64]
Liu T. 2019	46.31	8.05	49	62.64	12.27	53	9.1%	-1.55 [-1.99, -1.10]
McCombie A. 2023	2.82	2.38	35	3.23	3.04	33	9.0%	-0.15 [-0.63, 0.33]
Pan SQ 2022	6.84	1.24	25	7.82	1.56	25	8.8%	-0.68 [-1.26, -0.11]
Qing YF 2018	45.69	10.48	48	51.64	11.03	48	9.2%	-0.55 [-0.96, -0.14]
Zhang RL 2018	42.75	3.74	45	46.9	4.29	45	9.1%	-1.02 [-1.46, -0.58]
Zhu P. 2023	47.5	12.95	50	56.23	13.18	51	9.2%	-0.66 [-1.06, -0.26]
**Subtotal (95% CI)**			**355**			**358**	**72.7%**	**-0.46 [-1.24, 0.32]**
Heterogeneity: Tau² = 1.21; Chi² = 167.20, df = 7 (P < 0.00001); I² = 96%								
Test for overall effect: Z = 1.16 (P = 0.25)								
**8.2.3 Mindfulness Meditation**								
Chen YY 2021	50.82	9.29	38	56.37	6.82	38	9.1%	-0.67 [-1.14, -0.21]
**Subtotal (95% CI)**			**38**			**38**	**9.1%**	**-0.67 [-1.14, -0.21]**
Heterogeneity: Not applicable								
Test for overall effect: Z = 2.85 (P = 0.004)								
**Total (95% CI)**			**493**			**493**	**100.0%**	**-0.59 [-1.16, -0.02]**
Heterogeneity: Tau² = 0.89; Chi² = 181.22, df = 10 (P < 0.00001); I² = 94%								
Test for overall effect: Z = 2.02 (P = 0.04)								
Test for subgroup differences: Chi² = 3.51, df = 2 (P = 0.17), I² = 43.0%								

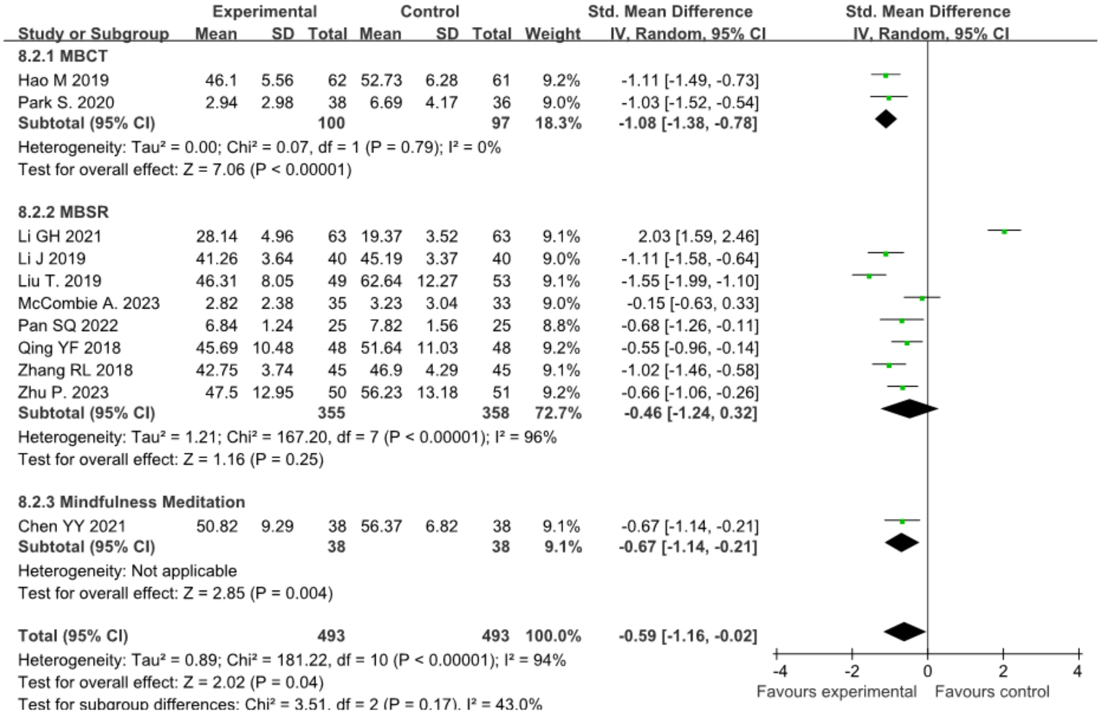

Positive thinking interventions

Study or Subgroup	Experimental Mean	Experimental SD	Experimental Total	Control Mean	Control SD	Control Total	Weight	Std. Mean Difference IV, Random, 95% CI
**7.2.1 Lymphoma**								
Chen YY 2021	50.82	9.29	38	56.37	6.82	38	9.1%	-0.67 [-1.14, -0.21]
Hao M 2019	46.1	5.56	62	52.73	6.28	61	9.2%	-1.11 [-1.49, -0.73]
Park S. 2020	2.94	2.98	38	6.69	4.17	36	9.0%	-1.03 [-1.52, -0.54]
Zhang RL 2018	42.75	3.74	45	46.9	4.29	45	9.1%	-1.02 [-1.46, -0.58]
Zhu P. 2023	47.5	12.95	50	56.23	13.18	51	9.2%	-0.66 [-1.06, -0.26]
**Subtotal (95% CI)**			**233**			**231**	**45.7%**	**-0.90 [-1.10, -0.71]**
Heterogeneity: Tau² = 0.00; Chi² = 4.00, df = 4 (P = 0.41); I² = 0%								
Test for overall effect: Z = 9.23 (P < 0.00001)								
**7.2.2 Nasopharyngeal carcinoma**								
Li GH 2021	28.14	4.96	63	19.37	3.52	63	9.1%	2.03 [1.59, 2.46]
Pan SQ 2022	6.84	1.24	25	7.82	1.56	25	8.8%	-0.68 [-1.26, -0.11]
**Subtotal (95% CI)**			**88**			**88**	**17.9%**	**0.68 [-1.98, 3.33]**
Heterogeneity: Tau² = 3.61; Chi² = 55.00, df = 1 (P < 0.00001); I² = 98%								
Test for overall effect: Z = 0.50 (P = 0.62)								
**7.2.3 Gynecological tumor**								
Li J 2019	41.26	3.64	40	45.19	3.37	40	9.0%	-1.11 [-1.58, -0.64]
Qing YF 2018	45.69	10.48	48	51.64	11.03	48	9.2%	-0.55 [-0.96, -0.14]
**Subtotal (95% CI)**			**88**			**88**	**18.2%**	**-0.82 [-1.37, -0.27]**
Heterogeneity: Tau² = 0.11; Chi² = 3.10, df = 1 (P = 0.08); I² = 68%								
Test for overall effect: Z = 2.91 (P = 0.004)								
**7.2.4 Thyroid cancer**								
Liu T. 2019	46.31	8.05	49	62.64	12.27	53	9.1%	-1.55 [-1.99, -1.10]
**Subtotal (95% CI)**			**49**			**53**	**9.1%**	**-1.55 [-1.99, -1.10]**
Heterogeneity: Not applicable								
Test for overall effect: Z = 6.83 (P < 0.00001)								
**7.2.5 Rectum**								
McCombie A. 2023	2.82	2.38	35	3.23	3.04	33	9.0%	-0.15 [-0.63, 0.33]
**Subtotal (95% CI)**			**35**			**33**	**9.0%**	**-0.15 [-0.63, 0.33]**
Heterogeneity: Not applicable								
Test for overall effect: Z = 0.61 (P = 0.54)								
**Total (95% CI)**			**493**			**493**	**100.0%**	**-0.59 [-1.16, -0.02]**
Heterogeneity: Tau² = 0.89; Chi² = 181.22, df = 10 (P < 0.00001); I² = 94%								
Test for overall effect: Z = 2.02 (P = 0.04)								
Test for subgroup differences: Chi² = 19.17, df = 4 (P = 0.0007), I² = 79.1%								

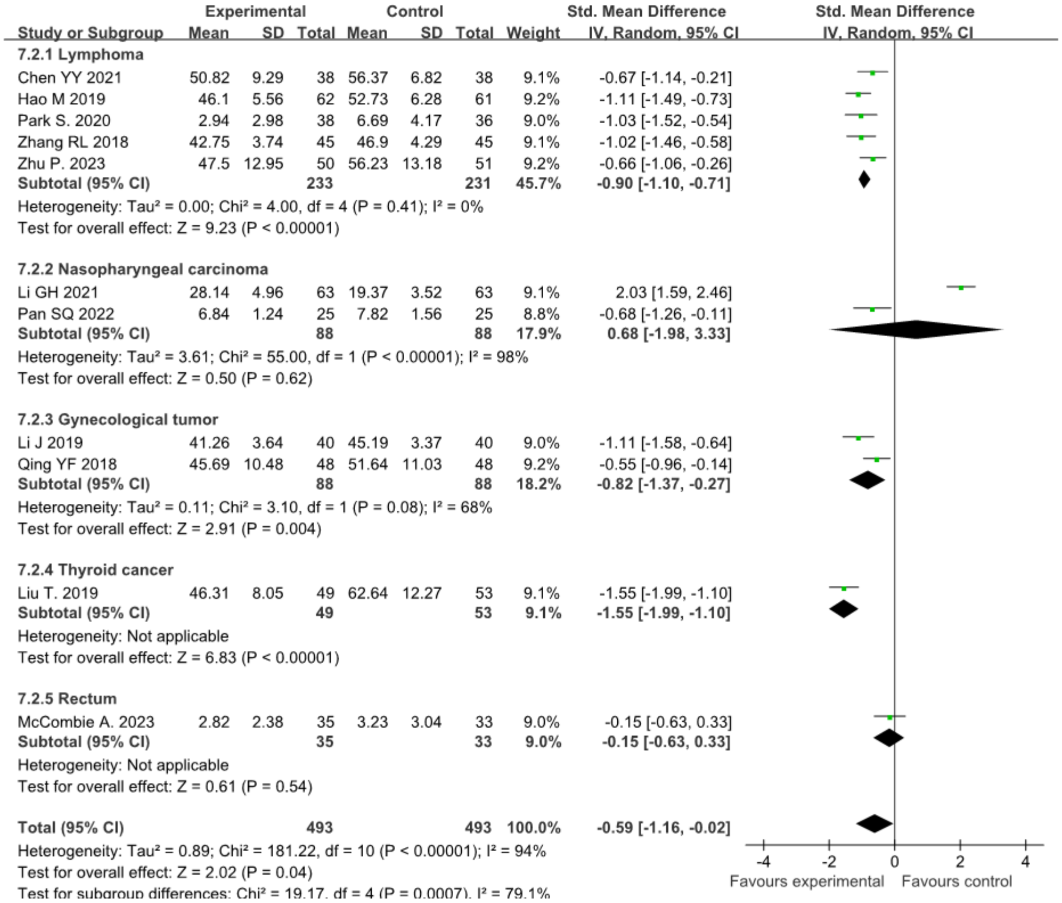

Tumour type

3. Quality of life subgroup analyses

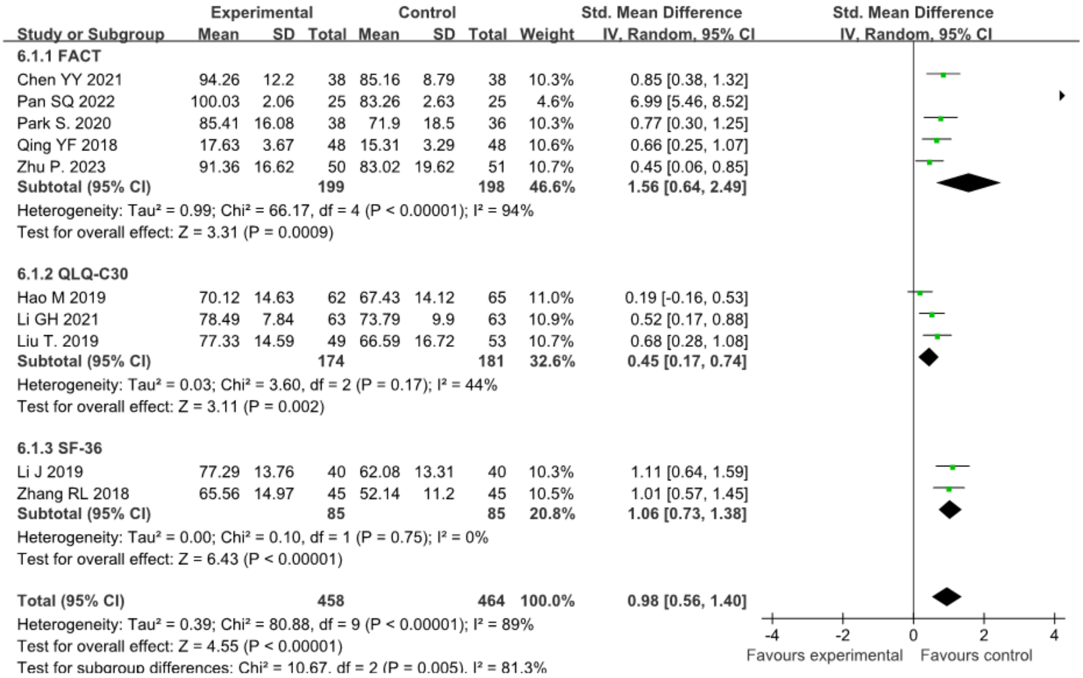

Subgroup analysis of assessment tools

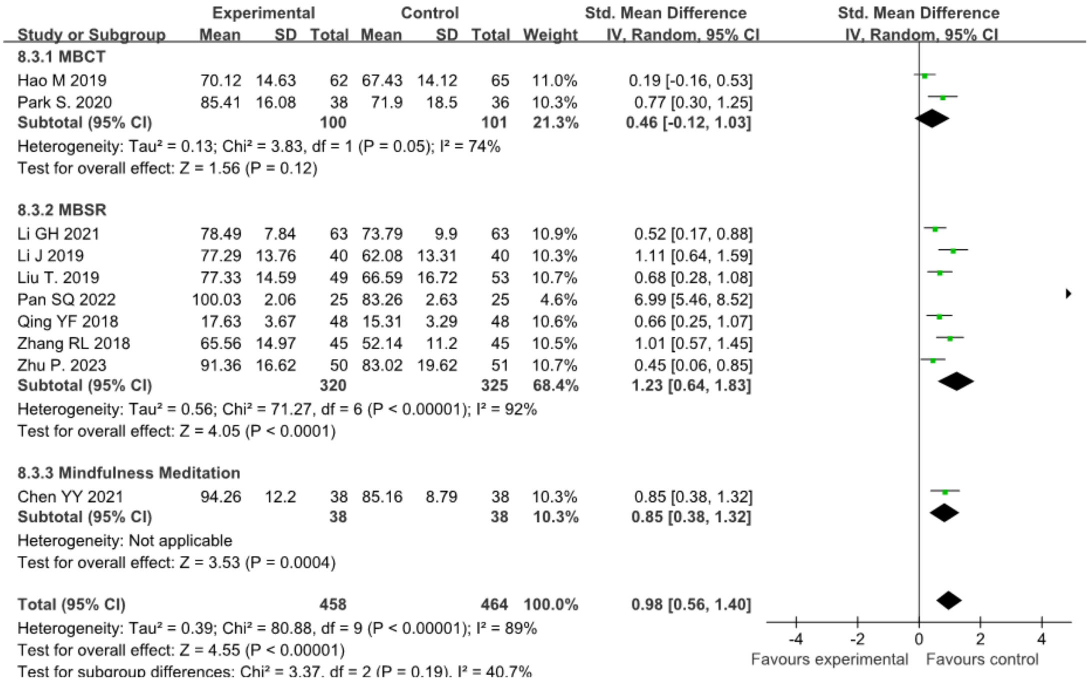

Positive thinking interventions

Study or Subgroup	Experimental Mean	Experimental SD	Experimental Total	Control Mean	Control SD	Control Total	Weight	Std. Mean Difference IV, Random, 95% CI
**7.3.1 Lymphoma**								
Chen YY 2021	94.26	12.2	38	85.16	8.79	38	10.3%	0.85 [0.38, 1.32]
Hao M 2019	70.12	14.63	62	67.43	14.12	65	11.0%	0.19 [-0.16, 0.53]
Park S. 2020	85.41	16.08	38	71.9	18.5	36	10.3%	0.77 [0.30, 1.25]
Zhang RL 2018	65.56	14.97	45	52.14	11.2	45	10.5%	1.01 [0.57, 1.45]
Zhu P. 2023	91.36	16.62	50	83.02	19.62	51	10.7%	0.45 [0.06, 0.85]
**Subtotal (95% CI)**			**233**			**235**	**52.8%**	**0.63 [0.32, 0.94]**
Heterogeneity: Tau² = 0.08; Chi² = 10.77, df = 4 (P = 0.03); I² = 63%								
Test for overall effect: Z = 4.00 (P < 0.0001)								
**7.3.2 Nasopharyngeal carcinoma**								
Li GH 2021	78.49	7.84	63	73.79	9.9	63	10.9%	0.52 [0.17, 0.88]
Pan SQ 2022	100.03	2.06	25	83.26	2.63	25	4.6%	6.99 [5.46, 8.52]
**Subtotal (95% CI)**			**88**			**88**	**15.6%**	**3.71 [-2.62, 10.05]**
Heterogeneity: Tau² = 20.57; Chi² = 65.00, df = 1 (P < 0.00001); I² = 98%								
Test for overall effect: Z = 1.15 (P = 0.25)								
**7.3.3 Gynecological tumor**								
Li J 2019	77.29	13.76	40	62.08	13.31	40	10.3%	1.11 [0.64, 1.59]
Qing YF 2018	17.63	3.67	48	15.31	3.29	48	10.6%	0.66 [0.25, 1.07]
**Subtotal (95% CI)**			**88**			**88**	**21.0%**	**0.87 [0.43, 1.31]**
Heterogeneity: Tau² = 0.05; Chi² = 2.00, df = 1 (P = 0.16); I² = 50%								
Test for overall effect: Z = 3.86 (P = 0.0001)								
**7.3.4 Thyroid cancer**								
Liu T. 2019	77.33	14.59	49	66.59	16.72	53	10.7%	0.68 [0.28, 1.08]
**Subtotal (95% CI)**			**49**			**53**	**10.7%**	**0.68 [0.28, 1.08]**
Heterogeneity: Not applicable								
Test for overall effect: Z = 3.32 (P = 0.0009)								
**Total (95% CI)**			**458**			**464**	**100.0%**	**0.98 [0.56, 1.40]**
Heterogeneity: Tau² = 0.39; Chi² = 80.88, df = 9 (P < 0.00001); I² = 89%								
Test for overall effect: Z = 4.55 (P < 0.00001)								
Test for subgroup differences: Chi² = 1.64, df = 3 (P = 0.65), I² = 0%								

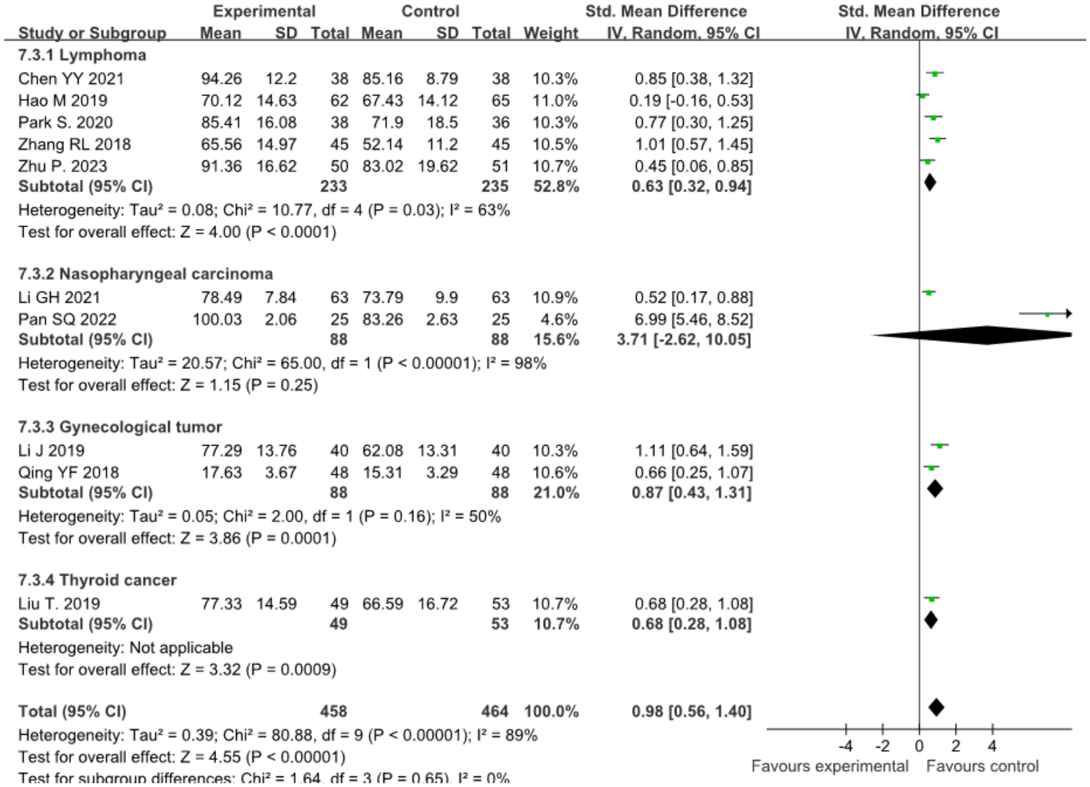

Tumour type
